# Common mental health disorders in adults with inflammatory skin conditions: nationwide population-based matched cohort studies in the UK

**DOI:** 10.1186/s12916-023-02948-x

**Published:** 2023-08-04

**Authors:** Alasdair D. Henderson, Elizabeth Adesanya, Amy Mulick, Julian Matthewman, Nhung Vu, Firoza Davies, Catherine H. Smith, Joseph Hayes, Kathryn E. Mansfield, Sinéad M. Langan

**Affiliations:** 1 Department of Non-Communicable Disease Epidemiology, London School of Hygiene & Tropical Medicine, Keppel Street, London, WC1E 7HT UK; 2 Patient and Public Advisory Panel, Skin Disease Epidemiology Research Group, London School of Hygiene & Tropical Medicine, London, UK; 3 King’s College London, St John’s Institute of Dermatology, London, UK; 4 Division of Psychiatry, University College London, London, UK; 5 Camden & Islington NHS Foundation Trust, London, UK

**Keywords:** Skin disease, Electronic health records, Anxiety, Depression

## Abstract

**Background:**

Psoriasis and atopic eczema are common inflammatory skin diseases. Existing research has identified increased risks of common mental disorders (anxiety, depression) in people with eczema and psoriasis; however, explanations for the associations remain unclear. We aimed to establish the risk factors for mental illness in those with eczema or psoriasis and identify the population groups most at risk.

**Methods:**

We used routinely collected data from the UK Clinical Practice Research Datalink (CPRD) GOLD. Adults registered with a general practice in CPRD (1997–2019) were eligible for inclusion. Individuals with eczema/psoriasis were matched (age, sex, practice) to up to five adults without eczema/psoriasis. We used Cox regression to estimate hazard ratios (HRs) and 95% confidence intervals (CIs) for hazards of anxiety or depression in people with eczema/psoriasis compared to people without. We adjusted for known confounders (deprivation, asthma [eczema], psoriatic arthritis [psoriasis], Charlson comorbidity index, calendar period) and potential mediators (harmful alcohol use, body mass index [BMI], smoking status, and, in eczema only, sleep quality [insomnia diagnoses, specific sleep problem medications] and high-dose oral glucocorticoids).

**Results:**

We identified two cohorts with and without eczema (1,032,782, matched to 4,990,125 without), and with and without psoriasis (366,884, matched to 1,834,330 without). Sleep quality was imbalanced in the eczema cohorts, twice as many people with eczema had evidence of poor sleep at baseline than those without eczema, including over 20% of those with severe eczema. After adjusting for potential confounders and mediators, eczema and psoriasis were associated with anxiety (adjusted HR [95% CI]: eczema 1.14 [1.13–1.16], psoriasis 1.17 [1.15–1.19]) and depression (adjusted HR [95% CI]: eczema 1.11 [1.1–1.12], psoriasis 1.21 [1.19–1.22]). However, we found evidence that these increased hazards are unlikely to be constant over time and were especially high 1-year after study entry.

**Conclusions:**

Atopic eczema and psoriasis are associated with increased incidence of anxiety and depression in adults. These associations may be mediated through known modifiable risk factors, especially sleep quality in people with eczema. Our findings highlight potential opportunities for the prevention of anxiety and depression in people with eczema/psoriasis through treatment of modifiable risk factors and enhanced eczema/psoriasis management.

**Supplementary Information:**

The online version contains supplementary material available at 10.1186/s12916-023-02948-x.

## Background

Inflammatory skin conditions such as eczema and psoriasis are common in adulthood; up to 10% worldwide have atopic eczema and up to 2% of UK adults have psoriasis [1–4]. Eczema (or atopic dermatitis) is characterised by recurrent skin lesions and intense itch [5] and psoriasis is a chronic inflammatory skin condition characterised by red scaly skin plaques [3].

Evidence indicates that eczema and psoriasis are associated with substantial comorbidity [2, 6–10] including mental health conditions [11–15]. However, most previous studies have not addressed temporality adequately and there is an absence of evidence regarding the effect of psoriasis on the risk of mental illness [16]. High-quality longitudinal evidence is therefore necessary to better understand why and when common mental illnesses occur in people with skin conditions.

Anxiety and depression are leading disease burdens worldwide [17] and are associated with increased morbidity and mortality [18, 19]. Evidence demonstrates that people with eczema/psoriasis are at increased risk of anxiety and depression [13–15, 20–25]. Plausible explanations for the association between eczema/psoriasis and anxiety/depression may include genetic risk factors [26–28], inflammation and living with a chronic disease [29, 30], related lifestyle factors (drinking [31, 32], smoking [33–35], reduced exercise [36–38]), comorbidities linked to eczema/psoriasis, stigma due to visible skin disease, or strong medications taken to treat the skin disease. The association between eczema/psoriasis and anxiety/depression may also differ by age, sex or over time due to changing prescribing and recording habits [39–41]. Many of these mechanisms have not been accounted for in previous research but are available in primary care records, such as comorbidities, steroid use and problems sleeping in people with eczema.

As both anxiety/depression and atopic eczema/psoriasis are common, a better understanding of this association and at-risk population groups could be of public health importance. Therefore, we conducted large, matched-cohort studies using routinely collected UK primary care records to better understand why and when new-onset anxiety or depression occurs in over 5 million UK adults with and without eczema/psoriasis.

## Methods

### Study population

We used primary care electronic health records from the UK’s Clinical Practice Research Datalink (CPRD GOLD) [42] to conduct two matched cohort studies (1. People with and without atopic eczema; 2. People with and without psoriasis) (1997–2019). CPRD GOLD (January 2020 build) includes over 18 million individuals and information on individual characteristics, morbidity-coded diagnoses, and prescriptions.

**Fig. 1:**
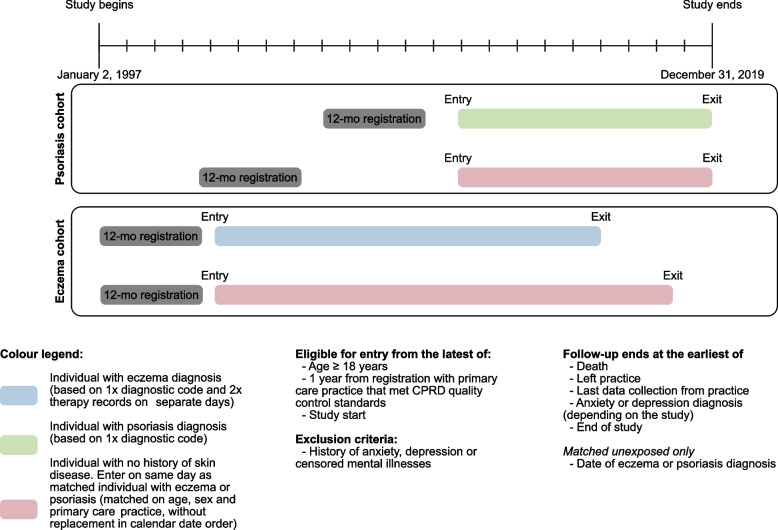
Schematic of the matched cohort study design. This schematic shows the study period and gives example of hypothetical study participants demonstrating their eligibility and study follow-up period

Adults (≥ 18 years) with at least 1 year of registration at a practice meeting CPRD quality-control standards were eligible for inclusion. We identified comparison cohorts of individuals randomly matched (without replacement) by age (± 5 years), sex, and general practice (to account for different practice coding patterns and regional differences) with up to five individuals without eczema/psoriasis (as appropriate) in calendar date order who entered the study on the same date as their matched exposed individual. The matched cohort design is shown in Fig. 1, with additional information in Additional file 1: Method S1. After matching, we developed four cohorts in total as we excluded individuals with anxiety (“anxiety cohorts”) or depression (“depression cohorts”) codes before cohort entry, or related diagnoses (e.g. severe mental illness).

### Atopic eczema/psoriasis

We identified individuals with atopic eczema based on a previously validated algorithm [27] requiring at least one diagnostic eczema code and at least two records for eczema therapies recorded on separate days. We identified individuals with psoriasis based on the earliest record of a diagnostic code for psoriasis [43, 44].

### Anxiety/depression

We identified anxiety or depression based on the earliest record of a diagnostic or symptom morbidity code for the relevant outcome recorded in CPRD [44, 45].

### Covariates

We used directed acyclic graphs (DAGs) to identify potential confounders or mediators of the relationship between eczema/psoriasis and anxiety/depression (Additional file 1: Fig. S1). Confounders included (1) age, sex, and calendar period (matching variables); (2) deprivation measured using quintiles of Carstairs deprivation index using 2011 census data [46]. The Carstairs index is calculated at the postcode level and is based on four Census variables: low social class, lack of car ownership, overcrowding and male unemployment; (3) specific comorbidities; asthma for people with eczema, and psoriatic arthritis for people with psoriasis; (4) Charlson comorbidity index (CCI) was calculated at baseline (including only comorbidities present before cohort entry) [47]. The CCI assigns weights from 1 (lowest) to 6 (highest) morbidity to 17 health conditions and we categorised individuals by their CCI score for comorbidities at baseline as low (0 points), intermediate (1–2 points), and high (> = 3 points); and (5) Ethnicity identified using a previously validated algorithm [48].

Possible mediators included (1) body mass index measured as close to study entry as possible (BMI, kg m^−2^, categorised as no evidence of obesity, obese class I (30–34.9), II (35–39.9), or III (40 +)); (2) harmful alcohol use (time-updated); and (3) smoking status (time-updated). In analyses with eczema as an exposure, we considered two additional mediators: (1) time-updated sleep problems defined based on morbidity coding (e.g. insomnia) or prescriptions for specific drugs used to manage sleep problems (e.g. zopiclone); and (2) high-dose oral glucocorticoid prescription (20 mg/day or more prednisolone equivalent dose) [2] as a time-updated 90-day risk window. In accordance with our DAG and due to uncertainty in the time of exposure recording in electronic health records [49, 50] we assumed that any record of a potential mediator occurred after exposure. Full covariate definitions are available in Additional file 1: Method S1 [2, 46–48, 51].

### Statistical analysis

We used Cox regression, stratified by matched set, with current age as the underlying timescale to estimate hazard ratios (HRs) and 95% confidence intervals (CI) for the association between eczema/psoriasis and anxiety/depression. We constructed three models: (1) minimally adjusted, implicitly adjusting for matched variables (age, sex, practice) and underlying age timescale; (2) additionally adjusted for confounders (deprivation, calendar period, comorbidity [asthma in eczema, psoriatic arthritis in psoriasis], CCI); and (3) additionally adjusted for potential mediators (harmful alcohol use, smoking, BMI, and, in eczema analyses only, sleep problems and high-dose oral glucocorticoids). We estimated incidence rates in the exposed from the data and in the unexposed population using inverse estimates from the confounder-adjusted model (Additional file 1: Method S1).

We conducted sensitivity analyses including ethnicity as a possible confounder (omitted in the main analysis due to missing data) and limiting to participants with a primary-care consultation either 1 or 3 years preceding cohort entry, to exclude practice non-attenders (Additional file 1: Method S1).

Our main analysis assumes that the hazard of eczema/psoriasis on anxiety/depression is constant over time. We tested this proportional hazard (PH) assumption through assessment of scaled Schoenfeld residuals [52]. As a supplementary analysis, we relaxed the PH assumption to allow the hazard of eczema/psoriasis on anxiety/depression to vary over time by including an interaction with a penalised spline with time using the ‘*pspline*’ function from the Survival package in R [53] (Additional file 1: Method S1).

### Secondary analyses

We extended the confounder-adjusted model to include an interaction between eczema/psoriasis and (1) age group; (2) sex. We also repeated the confounder-adjusted model redefining eczema/psoriasis exposure based on disease severity. We defined eczema and psoriasis severity as a time updated, monotonically increasing variable. We considered individuals to have mild disease by default [6, 11]. Eczema was considered moderate from a second potent topical corticosteroid or a first topical calcineurin inhibitor treatment, and severe from a first systemic immunosuppressant treatment, phototherapy code or referral for eczema. Psoriasis was mild by default and moderate/severe from the first record of systemic treatment, phototherapy, or a biologic therapy. Only two levels of psoriasis severity were included in line with previous research [54]. Further details on the definition of skin condition severity in our study are available in Additional file 1: Method S1.

Data management was performed in STATA version 16 [55] and R version 4.1.2 [56]. All analyses were performed in R version 4.1.2 and all code lists and analysis codes are available online [57] (Additional file 1: Method S1) [53, 55–63].

## Results

We initially identified 1,032,782 people with eczema matched to 4,990,125 without, and 366,884 with psoriasis matched to 1,834,330 without (Additional file 1: Fig. S2). After censoring people with the outcome of interest before cohort entry we analysed the four cohorts. We found that in each cohort, people with and without eczema/psoriasis had similar follow-up time and were broadly balanced in terms of age, sex, and deprivation (Table 1). We observed baseline imbalance in covariates between exposure groups for variables that are later included in regression modelling. For those in the anxiety cohorts, people with eczema/psoriasis were more likely to have a moderate/severe CCI score (37.3% with eczema versus 25.8% without; 29.3% with psoriasis versus 25.6% without), with similar patterns in the depression cohorts. More people with psoriasis had evidence of obesity (BMI > 30) (e.g. 21% with psoriasis versus 16% without in the anxiety cohort; 20% versus 15% in the depression cohort). There was missing data for ethnicity (~ 60%), smoking status (< 15%) and deprivation (< 5%). Missingness was broadly balanced between people with and without eczema/psoriasis (Additional file 1: Fig. S3 and Additional file 1: Tabs. S7-S8).

**Table 1:** Study participant characteristics at study entry for those with/without eczema or psoriasis, data are *n* (%) for categorical variables and median (IQR) for continuous variables

	**Eczema cohorts**				**Psoriasis cohorts**			
	**Anxiety cohort**		**Depression cohort**		**Anxiety cohort**		**Depression cohort**	
**Characteristic**	**Unexposed, *N* = 3,720,478**	**Eczema, *N* = 863,986**	**Unexposed, *N* = 3,228,8841**	**Eczema, *N* = 793,296**	**Unexposed, *N* = 1,365,258**	**Psoriasis, *N* = 310,081**	**Unexposed, *N* = 1,177,299**	**Psoriasis, *N* = 282,866**
**Follow-up time (years)**	4.5 (1.7–9.1)	4.9 (1.9–9.5)	4.4 (1.7–8.9)	4.7 (1.8–9.3)	5.7 (2.3–11.0)	5.4 (2.1–10.9)	5.6 (2.2–10.9)	5.3 (2.1–10.6)
**Sex (female)**	2,023,172 (54%)	484,720 (56%)	1,665,708 (52%)	431,807 (54%)	659,460 (48%)	155,059 (50%)	535,278 (45%)	136,554 (48%)
**Age**	38.6 (23.8–59.6)	39.9 (24.1–60.8)	37.3 (22.8–59.6)	39.3 (23.4–61.0)	43.4 (30.2–59.7)	44.0 (30.7–60.1)	43.0 (29.6–60.0)	43.8 (30.4–60.3)
18–39	1,936,683 (52%)	432,946 (50%)	1,729,474 (54%)	404,386 (51%)	603,064 (44%)	133,399 (43%)	529,725 (45%)	123,152 (44%)
40–49	456,639 (12%)	107,595 (12%)	365,872 (11%)	93,802 (12%)	223,800 (16%)	51,711 (17%)	184,885 (16%)	45,724 (16%)
50–59	416,104 (11%)	99,094 (11%)	338,787 (10%)	87,368 (11%)	201,742 (15%)	47,179 (15%)	168,318 (14%)	42,214 (15%)
60–69	403,886 (11%)	96,209 (11%)	348,075 (11%)	88,132 (11%)	168,935 (12%)	39,295 (13%)	146,033 (12%)	35,972 (13%)
70–79	317,043 (8.5%)	78,907 (9.1%)	283,161 (8.8%)	74,106 (9.3%)	114,154 (8.4%)	26,201 (8.4%)	101,858 (8.7%)	24,465 (8.6%)
80 +	190,123 (5.1%)	49,235 (5.7%)	163,515 (5.1%)	45,502 (5.7%)	53,563 (3.9%)	12,296 (4.0%)	46,480 (3.9%)	11,339 (4.0%)
**Carstairs deprivation quintile**								
1 (least deprived)	693,976 (19%)	164,020 (20%)	609,450 (20%)	153,083 (20%)	239,381 (18%)	54,491 (18%)	209,265 (19%)	50,577 (19%)
2	726,785 (20%)	169,436 (20%)	634,456 (20%)	156,814 (20%)	258,876 (20%)	58,095 (20%)	224,322 (20%)	53,369 (20%)
3	763,684 (21%)	177,947 (21%)	663,782 (21%)	163,294 (21%)	285,488 (22%)	64,962 (22%)	248,284 (22%)	59,633 (22%)
4	804,513 (22%)	185,506 (22%)	684,904 (22%)	167,523 (22%)	301,611 (23%)	68,634 (23%)	255,341 (23%)	61,631 (23%)
5 (most deprived)	616,149 (17%)	139,516 (17%)	529,446 (17%)	126,108 (16%)	222,091 (17%)	50,356 (17%)	187,961 (17%)	44,999 (17%)
Unknown	115,371	27,561	106,846	26,474	57,811	13,543	52,126	12,657
**Asthma diagnosis**	418,760 (11%)	194,298 (22%)	355,302 (11%)	175,497 (22%)	-	-	-	-
**Psoriatic arthritis**	-	-	-	-	8572 (0.6%)	3638 (1.2%)	6841 (0.6%)	3114 (1.1%)
**Charlson’s comorbidity index**								
Low	2,777,741 (75%)	542,649 (63%)	2,434,982 (75%)	503,973 (64%)	1,010,496 (74%)	219,105 (71%)	881,218 (75%)	202,700 (72%)
Moderate	803,045 (22%)	284,506 (33%)	681,188 (21%)	257,585 (32%)	305,143 (22%)	77,690 (25%)	256,072 (22%)	68,853 (24%)
Severe	139,692 (3.8%)	36,831 (4.3%)	112,714 (3.5%)	31,738 (4.0%)	49,619 (3.6%)	13,286 (4.3%)	40,009 (3.4%)	11,313 (4.0%)
**BMI**	25.2 (22.3–28.7)	25.4 (22.5–29.0)	25.1 (22.2–28.6)	25.2 (22.4–28.8)	25.6 (22.7–29.1)	26.1 (23.1–29.8)	25.5 (22.7–28.9)	26.0 (23.1–29.7)
Unknown	893,396	149,905	826,059	144,397	272,289	45,254	248,099	42,476
**Obesity (categorised)**								
No evidence of obesity	3,173,900 (85%)	717,101 (83%)	2,788,149 (86%)	667,059 (84%)	1,141,895 (84%)	245,756 (79%)	996,546 (85%)	226,971 (80%)
Obese class I (30–34.9)	366,497 (9.9%)	96,216 (11%)	301,783 (9.3%)	84,553 (11%)	151,030 (11%)	41,773 (13%)	125,007 (11%)	37,148 (13%)
Obese class II (35–39.9)	121,446 (3.3%)	33,420 (3.9%)	95,213 (2.9%)	28,048 (3.5%)	49,001 (3.6%)	14,862 (4.8%)	38,407 (3.3%)	12,628 (4.5%)
Obese class III (40 +)	58,635 (1.6%)	17,249 (2.0%)	43,739 (1.4%)	13,636 (1.7%)	23,332 (1.7%)	7690 (2.5%)	17,339 (1.5%)	6119 (2.2%)
**Harmful alcohol use**	96,328 (2.6%)	28,327 (3.3%)	72,753 (2.3%)	22,983 (2.9%)	41,845 (3.1%)	13,901 (4.5%)	31,626 (2.7%)	11,147 (3.9%)
**Smoking status**								
Non-smoker	1,858,380 (57%)	456,720 (56%)	1,642,116 (58%)	431,014 (58%)	662,851 (54%)	131,125 (45%)	580,837 (55%)	123,149 (46%)
Current smoker	642,990 (20%)	157,434 (19%)	523,575 (19%)	134,145 (18%)	255,508 (21%)	77,270 (26%)	210,144 (20%)	66,833 (25%)
Ex-smoker	687,105 (21%)	181,117 (22%)	575,059 (20%)	163,142 (22%)	273,966 (22%)	74,257 (25%)	229,228 (22%)	67,047 (25%)
Current or ex-smoker	97,395 (3.0%)	20,466 (2.5%)	76,982 (2.7%)	16,700 (2.2%)	36,229 (2.9%)	9614 (3.3%)	28,182 (2.7%)	7951 (3.0%)
Unknown	434,608	48,249	411,152	48,295	136,704	17,815	128,908	17,886
**Sleep problems**	257,752 (6.9%)	109,378 (13%)	190,349 (5.9%)	88,728 (11%)	-	-	-	-
**Severe eczema**								
None	3,720,478 (100%)	-	3,228,884 (100%)	-	1,365,258 (100%)	-	1,177,299 (100%)	-
Mild	-	638,977 (74%)	-	587,271 (74%)	-	303,327 (98%)	-	277,113 (98%)
Moderate	-	208,757 (24%)	-	190,798 (24%)				
Severe	-	16,252 (1.9%)	-	15,227 (1.9%)	-	6754 (2.2%)	-	5753 (2.0%)

We found twice as many people with eczema had evidence of sleeping problems than those without eczema. At baseline, in people with eczema, we found 13% of people in the anxiety cohort and 11% of people in the depression cohort had evidence of sleeping problems, compared to 6.8% and 5.9% of matched controls in these cohorts (Table 1). There was also a large difference in the proportion of sleep medication prescriptions by eczema severity (e.g. in the anxiety cohort; 6.4% of those with mild eczema had a relevant prescription, increasing to 17.9% of those with severe eczema) (Additional file 1: Fig. S4).

**Fig. 2:**
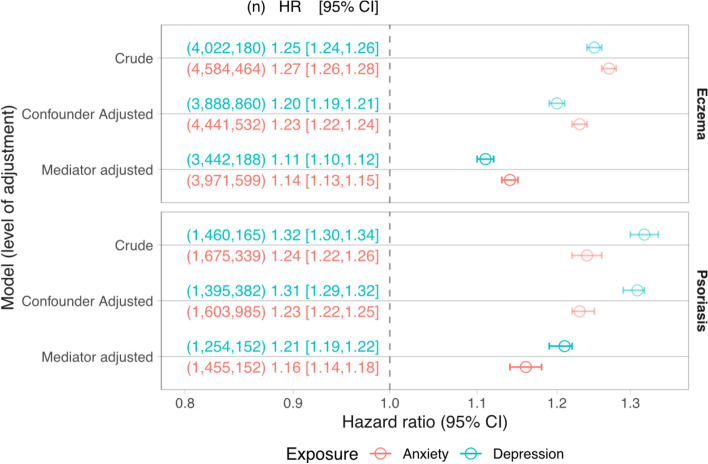
Hazard ratios for the association between eczema/psoriasis and anxiety or depression from stratified Cox models. Three models were developed for each exposure-outcome association with varying levels of adjustment. The crude model adjusted for matched set only (age, sex, practice). The confounder-adjusted model additionally adjusted for deprivation, calendar period, asthma (in eczema) or psoriatic arthropathy (in psoriasis), and Charlson comorbidity index. The mediator-adjusted model additionally adjusted for BMI (as evidence of obesity), smoking status, harmful alcohol use. Mediator-adjusted models in the eczema cohort additionally adjusted for sleep problems and immediate risk following a prescription of high-dose oral glucocorticoids. Dots; estimated HR. Lines; 95% CI. Text shows (the number of individuals in the model) HR, [95% CI]

We estimated that people with eczema and psoriasis had higher hazards of anxiety and depression than people without. For adults with eczema, the anxiety hazard was 1.23 times higher (95% CI: 1.22–1.24), and for depression 1.2 times higher (95% CI: 1.19–1.21) after adjusting for confounders (age, sex and practice, deprivation, calendar period, asthma and CCI). After further adjustment for potential mediators (alcohol, BMI, smoking, sleeping problems, high-dose oral glucocorticoids) the hazard ratios were 1.14 (95% CI: 1.13–1.15) for anxiety and 1.11 (95% CI: 1.1–1.12) for depression (Fig. 2, Additional file 1: Tab. S9).

In people with psoriasis, after adjusting for confounders, we found increased hazards for anxiety of 1.23 times (95% CI: 1.22–1.25), and 1.31 times for depression (95% CI: 1.29–1.32) compared to people without. After additionally adjusting for potential mediators, we found increased hazards of 1.16 times (95% CI: 1.14–1.18) for anxiety and 1.21 times (95% CI: 1.19–1.22) for depression (Fig. 2, Additional file 1: Tab. S9). All regression estimates are detailed in the appendix (Additional file 1: Tabs. S10–S13).

These relative differences correspond to a large burden of anxiety/depression in those with eczema/psoriasis. The largest estimated incidence rate difference was for those with psoriasis, with an estimated additional 456.9 (95% CI: 437.2–476.4) depression events per 100,000 person-years (Table 2).

**Table 2:** Absolute incidence rates and incidence rate differences (attributable risks) of anxiety and depression in people with and without eczema/psoriasis. Uncertainty intervals are the 2.5^th^ and 97.5^th^ percentiles from 5000 bootstrap samples

Outcome	Rate (exposed)^a^	Rate (unexposed)^ab^	Rate difference^a^
*Psoriasis*			
Anxiety	1274.8 (1262.7–1286.8)	1033.1 (1015.5–1051.1)	241.6 (226.5–256.7)
Depression	1946.4 (1930.5–1962.4)	1489.5 (1467.2–1512.3)	456.9 (437.2–476.4)
*Eczema*			
Anxiety	1468.8 (1460.7–1476.8)	1196.5 (1183.8–1209.2)	272.3 (261.6–283.3)
Depression	2095.7 (2085.3–2105.3)	1751.3 (1735.6–1766.9)	344.5 (330.3–357.8)

^a^Rate per 100,000 person-years

^b^Unexposed rate estimated as the rate (exposed) multiplied by the inverse hazard ratio from the confounder-adjusted model, because the unexposed cohort is not representative of the general population due to the matching in the study design

We found that more people with eczema/psoriasis had a consultation shortly before study entry. Over 10% of the comparator cohorts had not had a consultation in the 3 years before study entry (Additional file 1: Tab. S14); however, in sensitivity analyses, we limited the cohorts to people with consultations either 1 or 3 years before cohort entry and estimates were consistent with the main analysis (Additional file 1: Fig. S5).

### Time-varying hazards

**Fig. 3:**
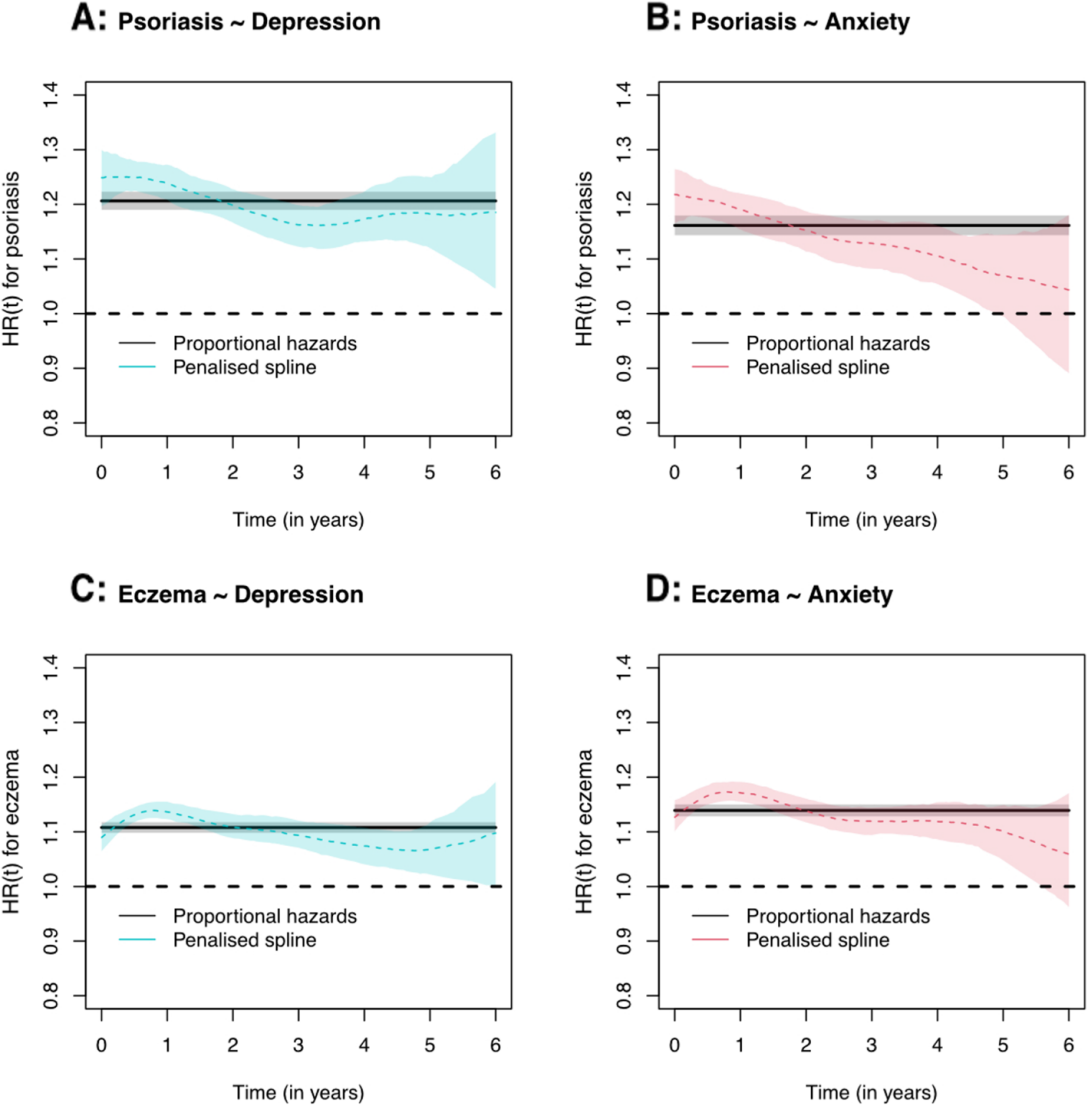
Estimated time-varying hazard ratios (HRs) for the association between inflammatory skin conditions and anxiety or depression. Red/blue dotted line and region, fully parametric penalised spline; black dotted line and region, the proportional hazards estimate and 95% CI from the relevant Cox model. Confidence intervals for the spline models show the 2.5 and 97.5 percentiles from 200 bootstrap iterations of the model. All models adjusted for age, sex, practice, deprivation, calendar period, asthma (in eczema) or psoriatic arthropathy (in psoriasis), and Charlson comorbidity index, BMI (as evidence of obesity), smoking status, harmful alcohol use (plus sleep problems and high dose oral glucocorticoid use in the eczema models)

We analysed whether the proportional hazards (PH) assumption had been met in our main analysis. We found deviations from time-invariant hazards ratios in all mediator-adjusted models (Additional file 1: Fig. S6). We therefore re-analysed the mediator and confounder adjusted Cox models, but additionally allowed the hazard ratio to vary over time (Additional file 1: Fig. S7). The aim was to reveal patterns of higher or lower anxiety/depression hazards over time in people with eczema/psoriasis. We found that the hazard ratio in all models for anxiety or depression was highest in the first year following diagnosis with eczema or psoriasis and declined thereafter (Fig. 3). From this model we estimated the mediator-adjusted hazard ratio at study entry, and at 1, 3, and 5 years of follow-up (Additional file 1: Tab. S15). These estimates show, for people with psoriasis, there are consistently high hazards for depression and that the hazard for anxiety ranged from a peak of 1.22 (95% CI: 1.17–1.26) at study entry (when eczema/psoriasis is diagnosed according to our definition), to 1.07 (95% CI: 0.99–1.14) after 5 years. For people with eczema, after 5 years, the mediator-adjusted hazards for anxiety (1.10; 95% CI: 1.05–1.15) and depression (1.07; 95% CI: 1.02–1.12) are lower than the proportional hazards estimates.

### Eczema/psoriasis severity

**Fig. 4:**
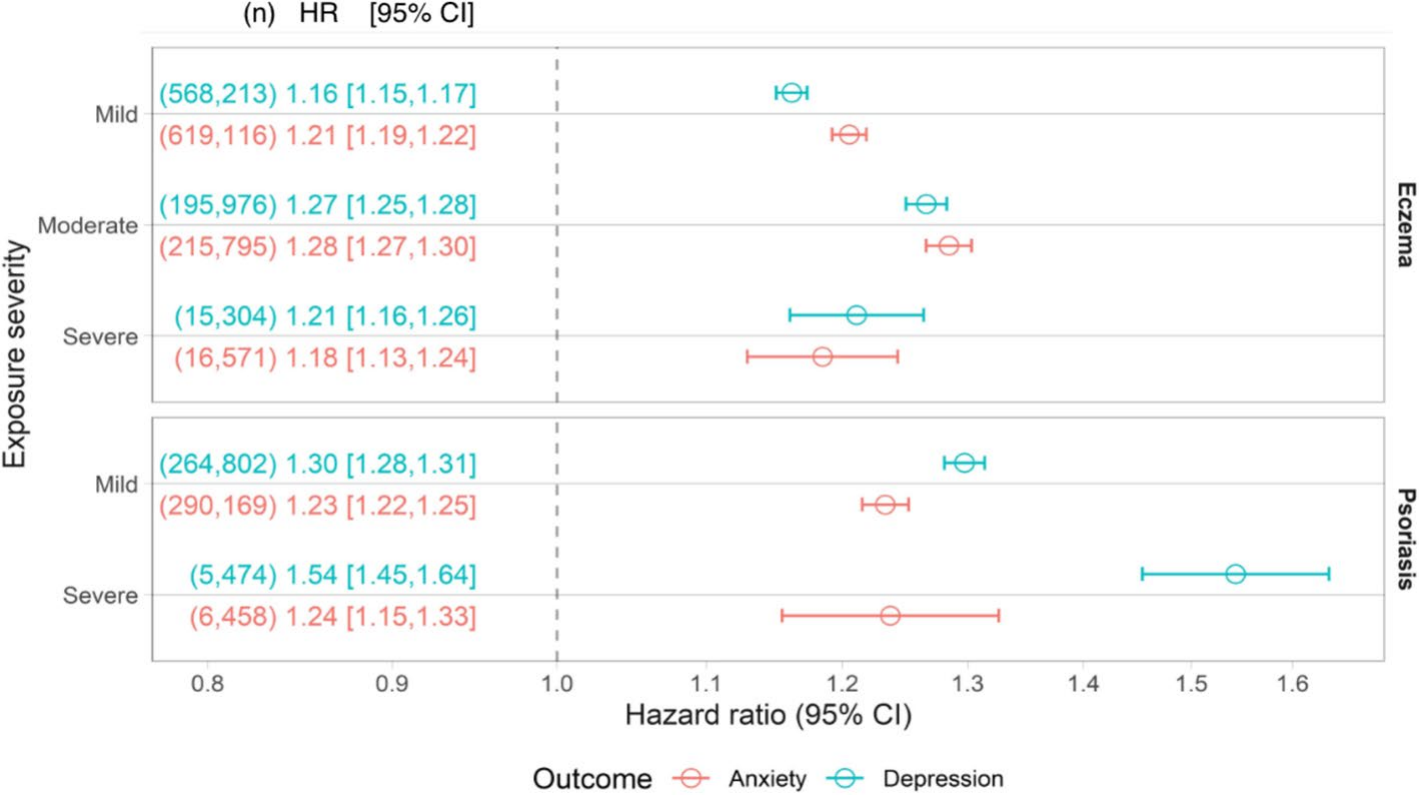
Hazard ratios for the association between eczema/psoriasis severity and anxiety or depression from stratified Cox models. Models were adjusted for confounders (age, sex, practice, deprivation, calendar period, asthma (in eczema) or psoriatic arthropathy (in psoriasis), and Charlson comorbidity index). Dots; estimated HR. Lines; 95% CI. Text shows (the number of individuals in the model) HR, [95% CI]

The effect of eczema/psoriasis on anxiety or depression varied slightly with eczema/psoriasis severity after adjusting for confounders. The noticeable exception was that, compared to people without psoriasis, the hazard for depression in people with severe psoriasis was larger (1.54; 95% CI: 1.45–1.64) than in the mild group (1.24; 95% CI: 1.15–1.33) (Fig. 4). There was some evidence that these hazards vary over time but that they were constant for the severe eczema/psoriasis group (Additional file 1: Fig. S8).

### Effect modification

We found evidence ($p$<0.001) that age modified the association between eczema/psoriasis and anxiety/depression. The confounder-adjusted association between eczema and anxiety/depression was lowest for those aged 18–39 (adjusted HR [95% CI]: anxiety 1.19 [1.17–1.2], depression 1.14 [1.13–1.16]), and highest for those aged 60–69 (adjusted HR [95% CI]: anxiety 1.29 [1.25–1.32], depression 1.27 [1.24–1.31]). There was no clear age pattern in the psoriasis cohort (Additional file 1: Fig. S9). We found evidence that sex modified the association between eczema/psoriasis and anxiety, but no evidence that sex had the same effect on the association with depression. Men had higher hazard ratios for anxiety than women in both the eczema and psoriasis cohorts. For example, men with psoriasis had 1.28 times the hazard for anxiety (95% CI [1.25–1.31]) compared to men without, and women had a hazard ratio of 1.21 (95% CI [1.19–1.23]) (Additional file 1: Fig. S9).

## Discussion

We found that adults with eczema or psoriasis had increased hazards of incident anxiety and depression. Even after adjusting for confounders and potential mediators, we found a 10–20% increased hazard for anxiety and depression in people with eczema or psoriasis compared to matched comparators. The largest observed association was for depression in people with psoriasis with an additional 456.9 cases per 100,000 person-years (95% CI: 437.2–476.4), and the hazard ratio for depression was especially large in people with severe psoriasis (1.54; 95% CI: 1.45–1.64).

These results may mask time trends however as we demonstrated that the hazard of anxiety or depression in people with eczema/psoriasis is unlikely to be constant over time. We found that the first year of follow-up in adults that met our eczema/psoriasis exposure definition is a period of elevated hazard for incident diagnoses of anxiety or depression in observational data.

We identified several population groups at higher risk of anxiety and depression, including older individuals (aged 60–69) with eczema and men with psoriasis. In terms of risk factors, it is possible that sleep problems are a key mediator of the association between eczema and anxiety/depression as these are particularly common in people with eczema compared to their matched comparators, and especially common in people with severe eczema (> 20% at baseline).

### Strengths and limitations

We identified large cohorts of adults with and without eczema/psoriasis and measured incident anxiety/depression diagnoses using prospectively collected population-based primary care data. Most people with eczema/psoriasis and anxiety/depression are managed in primary care [64] making these data appropriate to address our research question. However, our DAG shows that a causal estimate of the effect of eczema/psoriasis on anxiety or depression is beyond the scope of this study (Additional file 1: Fig. S1). The nature of routinely-collected data mean that we were limited in our ability to draw causal conclusions because (1) we had no information on some potentially key confounders (e.g. genetic risk) and mediators (e.g. physical activity) in primary care records; and (2) the likelihood of consulting in primary care (and entering our study) increases with both the eczema/psoriasis exposure and anxiety/depression outcome (leading to the possibility of collider bias). However, we found consistent results in our sensitivity analysis excluding participants who had not attended their practice within the 1 or 3 years prior to cohort entry.

One key limitation of our findings is that they are not generalisable to common mental disorders in childhood and adolescence. We designed the study to focus on the association between skin conditions and mental disorders and adulthood only because of key differences in diagnoses of anxiety and depression at younger ages due to differences in health-seeking behaviour [65].

Our study contains no information on people who manage their eczema/psoriasis outside of primary care and people may have eczema or psoriasis for a long period before it is recorded in practice records. If people with eczema/psoriasis were misclassified as not having eczema/psoriasis, our findings may underestimate the association with anxiety/depression. We were also unable to capture people who self-manage anxiety or depression (e.g. private counselling) so if people with eczema/psoriasis are more likely to report anxiety/depression at the GP and not self-manage our effect estimates will be an overestimate of the true association.

Primary care prescribing and coding patterns are heterogeneous and can change over time, often in line with relevant quality outcomes framework guidance [39–41] leading to temporal trends that are unlikely to have an underlying biological mechanism (e.g. increased hazard ratio for association between psoriasis and anxiety or depression since 2014). Cox models are often employed in survival analysis without sufficient investigation of the key underlying assumption, that the hazard ratio is constant over time [66, 67]. We presented an alternative analysis to capture the non-constant hazard ratio. This approach revealed a period of higher hazard for anxiety or depression up to 1 year following eczema/psoriasis diagnosis. Possible explanations include (1) ascertainment bias where diagnosis of a second condition (e.g. depression) is more likely when somebody attends their practice for a primary condition (e.g. psoriasis); or (2) first year adapting to a new chronic condition causes anxiety or depression. In this study, we cannot differentiate between these two potential explanations.

This is the first study, to our knowledge, to incorporate sleep problems in a study of mental health conditions in those with eczema in routine data. However, some key confounders were only partially captured in our study, including BMI and smoking status, deprivation (which is only partially captured by the Carstairs index of deprivation) [46], and ethnicity. To limit missing BMI and smoking status, we took included future information. Ethnicity data were often missing, so we could only include ethnicity in sensitivity analyses, with minimal change to our estimates (Additional file 1: Fig. S7). Missing ethnicity data is a serious limitation and further research is needed to investigate possibly important health inequalities.

There are several variables that have not been included in this study that may affect the relationship between inflammatory skin conditions and mental health disorders. Some psychiatric medications may play a role in this association, notably lithium and beta blockers. However, since our outcome was new onset anxiety/depression, diagnosis should precede psychiatric medications. Additional potential unmeasured covariates identified through discussion with our Public and Patient Involvement panel include a measure of sleep quality (instead of diagnoses of, and prescriptions for, problems sleeping), location on the body and visibility of the skin disease, time of year, and family support. We observed covariate imbalance at baseline between the exposed and unexposed populations, notably with comorbidities. We adjusted for these variables in regression analysis and consistently found a higher hazard for mental illness in those with eczema/psoriasis. Alternative methods including disease risk score matching can achieve better balance between covariates [68]. However, accurately modelling disease risk score is challenging [69] and beyond the scope of this work, and in some cases this method is not superior to regression adjustment [70]. Future research could use these methods to explore whether observed differences in our study are explained by unmeasured confounding.

We did not assess the possible additive effect of exposure to both eczema and psoriasis. We described the number of people meeting both psoriasis and eczema definitions in our cohorts and found that 7.75% of individuals with eczema also had a psoriasis diagnosis. A larger proportion (21.8%) of the psoriasis cohort also had an eczema diagnosis, but the majority had psoriasis only (Additional file 1: Tab. S16). Further research could explore the interaction between these skin conditions on the risk of mental illness; however, our study was not designed or powered to robustly estimate these effects.

We aimed to identify key risk factors for the association between eczema/psoriasis and anxiety/depression. We found evidence that problems sleeping were far more common in those with eczema than matched comparators and we observed interesting changes in hazard ratios between the confounder- and mediator-adjusted models. However, we cannot formally compare these estimates because of the differing covariates in the models and a formal mediation analysis is necessary [71].

### Comparison with other studies

This research follows previous work identifying eczema and psoriasis as possible risk factors for anxiety or depression [11]. However, most existing research has been through cross-sectional [20, 22, 23, 72] or case–control [14] designs, or cohort studies that adjusted for restricted confounders/mediators [25, 73] or in small samples [74]. Our study extends a previous large, matched cohort study [11] in two crucial ways: firstly, we considered psoriasis in addition to eczema, facilitating comparisons between different visible skin diseases and revealing especially high hazards for depression in those with severe psoriasis. Secondly, we included key variables that were absent in previous research, namely asthma, psoriatic arthritis, and sleep problems.

One recent genetic study using UK Biobank identified no causal mechanism for eczema to cause anxiety or depression [75]. There is, however, evidence from genetic research that inflammation (increased interleukin 6 activity) is a risk factor for anxiety/depression [30] and suicidality [76], and higher polygenic risk scores for depression in those with psoriasis, and vice versa [26]. Further research is needed in this area however our research demonstrates that key modifiable mediators (especially problems sleeping) could help explain any observed clinical association.

### Explanations and implications

Our study demonstrates that people with eczema or psoriasis are more likely to have anxiety or depression after their skin disease diagnosis. This risk is particularly elevated in the first year, potentially reflecting ascertainment bias in primary care records or indicating a risk window where individuals are more likely to develop anxiety/depression that primary care doctors should be aware of following an eczema/psoriasis diagnosis. The reduced hazard ratio over time may also be explained by improved skin condition treatment and severity over time; however, we found similar patterns over time in the “mild” eczema and psoriasis groups in our severity analysis (Additional file 1: Fig. S8). Further research is needed in data where skin condition severity over time can be monitored more accurately.

Factors that may explain the association are complex and might include alcohol intake, obesity, and (in people with eczema) problems sleeping. Mediators, such as sleeping problems or repeated high-dose steroid use, are therefore possible points of intervention for better mental health outcomes in people with skin disease. Future research into this problem should aim to quantify the relative contributions of these mediators and adjust for further factors that were not included in this study. Our results suggest that incorporating specific strategies to address problems with insomnia in atopic eczema may be helpful in relation to mental health problems.

## Conclusions

Eczema and psoriasis in adults are associated with increased incidence of anxiety and depression. Our study indicates that at least part of this risk is mediated through modifiable risk factors, including sleeping problems in people with eczema. Our findings highlight potential opportunities for the prevention of anxiety and depression in people with eczema/psoriasis through treatment of modifiable risk factors and enhanced eczema/psoriasis management [77].

### Supplementary Information

**Additional file 1: Methods S1.** additional information on cohort design, statistical methods and computer software [77–79]. **Table S1.** Full baseline characteristics of people with and without eczema at cohort entry for anxiety/depression. **Table S2.** Full baseline characteristics of people with and without psoriasis at cohort entry for anxiety/depression. **Table S3.** Data availability for quintiles of the Carstairs index of deprivation. **Table S4.** Characteristics of time updated covariates throughout the entire study period. **Table S5.** Person years under follow up in adults with and without eczema by individual-level characteristics. **Table S6.** Person years under follow up in adults with and without psoriasis by individual-level characteristics. **Table S7.** Baseline characteristics of those with and without complete data, comparing adults with and without eczema. **Table S8.** Baseline characteristics of those with and without complete data, comparing adults with and without psoriasis. **Table S9.** Hazard ratios from Cox regression models for the association between inflammatory skin conditions and anxiety or depression. **Table S10.** All parameter estimates from Cox modelling of the association between *eczema and anxiety*. **Table S11.** All parameter estimates from Cox modelling of the association between *eczema and depression*. **Table S12.** All parameter estimates from Cox modelling of the association between *psoriasis and anxiety*. **Table S13.** All parameter estimates from Cox modelling of the association between *psoriasis and depression*. **Table S14.** Analysis of individuals with/without a consultation with their GP either 1-year or 3-years before study entry. **Table S15.** Estimates of the time-varying hazard ratios for the association between eczema/psoriasis and anxiety/depression at multiple time-points following study entry. **Table S16.** Number of people exposed to eczema, psoriasis or both skin conditions in the overall exposed cohorts. **Figure S1.** Directed acyclic graph for the association between eczema and anxiety or depression. **Figure S2.** Participant flowcharts. **Figure S3.** Percentage of missing data in the both the eczema and psoriasis cohorts. **Figure S4.** Percentage of individuals with eczema at study entry with a record for a diagnosis or prescription for sleeping problems by varying levels of eczema severity. **Figure S5.** Sensitivity analyses: Hazard ratios for the association between eczema/psoriasis. and anxiety/depression from stratified Cox models. **Figure S6.** Schoenfeld residuals to test the proportional hazards assumption for the association between eczema /psoriasis and anxiety/depression. **Figure S7.** Exploring non-constant hazard ratios for the association between inflammatory skin conditions and anxiety or depression by including a linear interaction with time. **Figure S8.** Schoenfeld residuals to check the proportional hazards assumption for the association between eczema/psoriasis *severity* and anxiety/depression. **Figure S9.** Estimated hazard ratios for the total effect of inflammatory skin conditions on anxiety or depression, modified by several pre-specified effect modifiers.

## Data Availability

No additional unpublished data are available as this study used existing data from the UK CPRD electronic health record database, which is only accessible to researchers with protocols approved by the CPRD’s Research Data Governance (RDG) Process. All analysis computer code and code lists are available via GitHub (see Methods). All code is shared without investigator support.

## Abbreviations

BMI: Body mass index
CCI: Charlson’s comorbidity index
CPRD: Clinical Practice Research Datalink
DAG: Directed acyclic graph
CI: Confidence interval
HR: Hazard ratio
PH: Proportional hazards
SMI: Severe mental illness
