# Supplementary material for: Common mental health disorders in adults with inflammatory skin conditions: nationwide population-based matched cohort studies in the UK
Source: BMC Med. 2023 Aug 4;21:285. doi: 10.1186/s12916-023-02948-x (PMC10403838; doi:10.1186/s12916-023-02948-x)
Supplement: Supplementary file 1 — Additional file 1: Methods S1. additional information on cohort design, statistical methods and computer software [77–79]. Table S1. Full baseline characteristics of people with and without eczema at cohort entry for anxiety/depression. Table S2. Full baseline characteristics of people with and without psoriasis at cohort entry for anxiety/depression. Table S3. Data availability for quintiles of the Carstairs index of deprivation. Table S4. Characteristics of time updated covariates throughout the entire study period. Table S5. Person years under follow up in adults with and without eczema by individual-level characteristics. Table S6. Person years under follow up in adults with and without psoriasis by individual-level characteristics. Table S7. Baseline characteristics of those with and without complete data, comparing adults with and without eczema. Table S8. Baseline characteristics of those with and without complete data, comparing adults with and without psoriasis. Table S9. Hazard ratios from Cox regression models for the association between inflammatory skin conditions and anxiety or depression. Table S10. All parameter estimates from Cox modelling of the association between eczema and anxiety. Table S11. All parameter estimates from Cox modelling of the association between eczema and depression. Table S12. All parameter estimates from Cox modelling of the association between psoriasis and anxiety. Table S13. All parameter estimates from Cox modelling of the association between psoriasis and depression. Table S14. Analysis of individuals with/without a consultation with their GP either 1-year or 3-years before study entry. Table S15. Estimates of the time-varying hazard ratios for the association between eczema/psoriasis and anxiety/depression at multiple time-points following study entry. Table S16. Number of people exposed to eczema, psoriasis or both skin conditions in the overall exposed cohorts. Figure S1. Directed acyclic graph for the association betw [file 12916_2023_2948_MOESM1_ESM.pdf]

Supplementary materials

Common mental health disorders in adults with inflammatory skin conditions: nationwide population based matched cohort studies in the UK

Authors: Alasdair D Henderson[1], Elizabeth Adesanya[1], Amy Mulick[1], Julian Matthewman[1], Nhung Vu[2], Firoza Davies[2], Catherine H Smith[3], Joseph Hayes[4], Kathryn E Mansfield[1], Sinéad M Langan[1]

Affiliations:
[1] Department of Non-Communicable Disease Epidemiology, London School of Hygiene & Tropical Medicine, London, UK
[2] Patient and Public Advisory Panel, Skin Disease Epidemiology Research Group, London School of Hygiene & Tropical Medicine, London, UK
[3] King's College London, St John's Institute of Dermatology, London, United Kingdom
[4] Division of Psychiatry, University College London, London, UK
Camden & Islington NHS Foundation Trust, London, UK

LIST OF TABLES 3

LIST OF FIGURES 4

SUPPLEMENTARY METHODS 6

Data source & study population: 6

Individuals could contribute person-time in the unexposed and exposed cohorts 6

Matched cohort design 6

Exposure definition 6

Covariates 7

Statistical analysis 8

Sensitivity analysis 9

Computer software 9

References 9

SUPPLEMENTARY TABLES 10

SUPPLEMENTARY FIGURES 29

List of tables

Table 1: Full baseline characteristics of people with and without eczema at cohort entry for anxiety/depression. Data are N (%) if categorical, and median (IQR) if continuous. 10

Table 2: Full baseline characteristics of people with and without psoriasis at cohort entry for anxiety/depression. Data are N (%) if categorical, and median (IQR) if continuous. 11

Table 3: Data availability for quintiles of the Carstairs index of deprivation. Patient-level data was used where available, practice-level if not. Data for all practices were not available from record linkage. 12

Table 4: Characteristics of time updated covariates throughout the entire study period (1997-2020). This table shows the number (%) of participants with a record at any point during their eligible follow up.......... 12

*Table 5: Person years under follow up in adults **with and without eczema** by individual-level characteristics. Values are person-years at risk (%)* 13

*Table 6: Person years under follow up in adults **with and without psoriasis** by individual-level characteristics. Values are person-years at risk (%)* 15

*Table 7: Baseline characteristics of those with and without complete data, comparing adults **with and without eczema**. Value are number of individuals (%). Variable with missingness are Carstairs index of deprivation, BMI, Smoking status* 17

*Table 8: Baseline characteristics of those with and without complete data, comparing adults **with and without psoriasis**. Value are number of individuals (%). Variable with missingness are Carstairs index of deprivation, BMI, Smoking status* 18

Table 9: Hazard ratios from Cox regression models for the association between inflammatory skin conditions and anxiety or depression. Full parameter estimates from all models are available in the supplementary materials (Table 10 - Table 13).......... 21

Table 10: All parameter estimates from Cox modelling of the association between eczema and anxiety. Three models were developed for each exposure-outcome association with varying levels of adjustment. The crude model adjusted for matched set only (age, sex, GP). The confounder adjusted model additionally adjusted for deprivation, calendar period, asthma (in eczema) or psoriatic arthropathy (in psoriasis), and Charlson comorbidity index. The mediator adjusted model additionally adjusted for BMI (as evidence of obesity), smoking status, harmful alcohol use. Mediator adjusted models in the eczema cohort additionally adjusted for sleep problems and immediate risk following a prescription of high dose oral glucocorticoids. Note: these estimates are presented for completeness and to aid reproducibility, The effect estimates for variables other than the primary exposure (eczema/psoriasis) carry no clinical relevance. ... 22

*Table 11: All parameter estimates from Cox modelling of the association between **eczema and depression**. Three models were developed for each exposure-outcome association with varying levels of adjustment. The crude model adjusted for matched set only (age, sex, GP). The confounder adjusted model additionally adjusted for deprivation, calendar period, asthma (in eczema) or psoriatic arthropathy (in psoriasis), and Charlson comorbidity index. The mediator adjusted model additionally adjusted for BMI (as evidence of obesity), smoking status, harmful alcohol use. Mediator adjusted models in the eczema cohort additionally adjusted for sleep problems and immediate risk following a prescription of high dose oral glucocorticoids Note: these estimates are presented for completeness and to aid reproducibility, The effect estimates for variables other than the primary exposure (eczema/psoriasis) carry no clinical relevance.* ... 23

*Table 12: All parameter estimates from Cox modelling of the association between **psoriasis and anxiety**. Three models were developed for each exposure-outcome association with varying levels of adjustment. The crude model adjusted for matched set only (age, sex, GP). The confounder adjusted model additionally adjusted for deprivation, calendar period, asthma (in*

eczema) or psoriatic arthropathy (in psoriasis), and Charlson comorbidity index. The mediator adjusted model additionally adjusted for BMI (as evidence of obesity), smoking status, harmful alcohol use. Note: these estimates are presented for completeness and to aid reproducibility, The effect estimates for variables other than the primary exposure (eczema/psoriasis) carry no clinical relevance. 24

*Table 13: All parameter estimates from Cox modelling of the association between **psoriasis and depression**. Three models were developed for each exposure-outcome association with varying levels of adjustment. The crude model adjusted for matched set only (age, sex, GP). The confounder adjusted model additionally adjusted for deprivation, calendar period, asthma (in eczema) or psoriatic arthropathy (in psoriasis), and Charlson comorbidity index. The mediator adjusted model additionally adjusted for BMI (as evidence of obesity), smoking status, harmful alcohol use. Note: these estimates are presented for completeness and to aid reproducibility, The effect estimates for variables other than the primary exposure (eczema/psoriasis) carry no clinical relevance.* 26

Table 14: Analysis of individual with/without a consultation with their GP either 1-year or 3-years before study entry, comparing those with and without the inflammatory skin condition of interest 28

Table 15: Estimates of the time-varying hazard ratios for the association between eczema/psoriasis and anxiety/depression at multiple time-points following study entry. All models adjusted for age, sex, GP, deprivation, calendar period, asthma (in eczema) or psoriatic arthropathy (in psoriasis), and Charlson comorbidity index, BMI (as evidence of obesity), smoking status, harmful alcohol use (plus sleep problems and high dose oral glucocorticoid use in the eczema models). Confidence intervals for the spline models show the 2.5 and 97.5 percentiles from 200 bootstrap iterations of the model. 28

Table 16: Number of people meeting our eczema algorithm, psoriasis definition or both in the overall exposed cohorts 28

List of Figures

Figure 1: Directed acyclic graph for the association between eczema or psoriasis with anxiety or depression. White and blue circles, variables adjusted for in this study. Grey circles, unobserved variables not included in models (except ethnicity which is included in a sensitivity analysis). For simplicity we have not displayed possible links between covariates (e.g., BMI and harmful alcohol use) 29

*Figure 2: Participant flowcharts. * exposure algorithms: eczema (based on 1x diagnostic code and 2x therapy records on separate days); psoriasis (based on 1x diagnostic code)* 30

Figure 3: Percentage of missing data in the both the eczema (A) and psoriasis (B) cohorts, comparing those with and without eczema/psoriasis 31

Figure 4: Percentage of individuals with eczema at study entry with a record for a diagnosis or prescription for sleeping problems by varying levels of eczema severity. (A and B) percentage of the cohort with a record of a sleep problems diagnosis or sleep-specific prescriptions. Size of bubble is scaled by number of codes in each group. Colour and label show the percentage with a code at baseline. (B) Percentage of cohort with specific treatments/diagnoses by eczema severity. All codes with at least 0.1% prevalence over the study period are displayed 32

Figure 5: Sensitivity analyses: Hazard ratios (HRs) for the association between inflammatory skin conditions. and anxiety or depression from stratified Cox models. Three models were developed for each exposure-outcome association with varying levels of adjustment. The crude model adjusted for matched set only (age, sex, GP). The confounder adjusted model additionally adjusted for deprivation, calendar period, asthma (in eczema) or psoriatic arthropathy (in psoriasis), and Charlson comorbidity index. The mediator adjusted model additionally adjusted for BMI (as evidence of obesity), smoking status, harmful alcohol use. Mediator adjusted models in the eczema cohort additionally adjusted for sleep problems and immediate risk following a

prescription of high dose oral glucocorticoids. Dots; estimated HR. Lines; 95% CI. Text shows (the number of individuals in the model) HR, [95% CI]. 33

Figure 6: Checking the proportional hazards assumption for the association between eczema /psoriasis and anxiety/depression. Left column, scaled Schoenfeld residuals from the mediator-adjusted Cox model. Right column, zoomed in on the smoothed spline through the scaled Schoenfeld residuals in left-hand panel, which is equivalent to a time varying HR from the model. Green solid and dashed lines, median estimate and 95% CI; Blue dotted line and region, the corresponding proportional HR from the Cox model and 95% CI. 34

Figure 7: Exploring non-constant hazard ratios (HRs) for the association between inflammatory skin conditions and anxiety or depression by including a linear interaction with time. The assumption that time acts in a linear fashion is strong and lead to extreme results in later periods of follow up. These results were improved on by including a non-linear interaction with time in the main paper 35

Figure 8: Checking the proportional hazards assumption for the association between ***eczema/psoriasis severity and anxiety/depression****. Each row shows estimates from a single Cox model. Plots show a smoothed spline through the exponential of the scaled Schoenfeld residuals which is equivalent to a time varying HR from the model. Green solid and dashed lines, median estimate and 95% CI; Blue dotted line and region, the corresponding proportional HR from the Cox model and 95% CI. All models adjusted for age, sex, GP, deprivation, calendar period, asthma (in eczema) or psoriatic arthropathy (in psoriasis), and Charlson comorbidity index, BMI (as evidence of obesity), smoking status, harmful alcohol use (plus sleep problems and high dose oral glucocorticoid use in the eczema models).* 36

Figure 9: Estimated hazard ratios for the total effect of inflammatory skin conditions on anxiety or depression, modified by several pre-specified effect modifiers. HRs and 95% confidence intervals are derived from linear combinations of model parameters (confounder) adjusted for age, sex, GP, deprivation, calendar period, asthma (in eczema) or psoriatic arthropathy (in psoriasis), and Charlson comorbidity index. Dots; estimated HR. Lines; 95% CI 37

Supplementary methods

Data source & study population:

CPRD is a database of routinely collected primary care electronic health records from a sample of general practices (GPs) that represents around 7% of the UK population are broadly representative of the UK general population. The CPRD GOLD (January 2020 build) includes over 18 million individuals and information on individual demographics, morbidity-coded diagnoses, and prescriptions.

Individuals could contribute person-time in the unexposed and exposed cohorts

Individuals without eczema/psoriasis entered their respective cohorts on the same date as the matched exposed individual. Individuals without eczema/psoriasis that later met the exposure definition were initially eligible for the unexposed comparison cohort until that date. People included in the comparison (unexposed) cohorts who have a subsequent record of a diagnostic code for eczema/psoriasis (recorded in primary care) after cohort entry will be censored from the comparison cohort and become eligible for inclusion in the relevant skin disease cohort (i.e. people included in the atopic eczema comparison cohort who have a record of an atopic eczema diagnosis after cohort entry will no longer be followed up in the comparison cohort, but, will be considered for inclusion in the atopic eczema cohort, and similarly for the psoriasis cohort).

Matched cohort design

Individuals were eligible for cohort entry from the latest of: study start (January 1997), one year after GP registration (to allow time to capture existing diagnoses in medical records), 18th birthday, or date their practice met CPRD quality-control standards. Individuals with eczema/psoriasis entered cohorts from the latest of the date they met eligibility criteria or atopic eczema/psoriasis definitions. Individuals without eczema/psoriasis entered on the same date as their matched individual with eczema/psoriasis. All individuals were followed until the earliest of: anxiety or depression diagnosis (outcome), death, end of registration, no further data from practice, end of study (Dec 2019), or, in the matched comparison group, a record of a diagnostic code for atopic eczema/psoriasis (as appropriate). After matching, we excluded individuals with any mental illness diagnoses relevant to the specific outcome under investigation diagnosed before cohort entry or related diagnoses (e.g., severe mental illness) (Figure S1, Figure S2).

Exposure definition

Eczema

The algorithms we used to identify eczema exposures was based on a combination of at least one diagnostic code and at least two records for skin disease therapies recorded on separate days [77]. Records for skin disease therapies included: 1) records of phototherapy (Read codes in primary care); and 2) primary care prescriptions for relevant topical or oral drugs (i.e., topical emollients, corticosteroids or calcineurin inhibitors, oral glucocorticoids, azathioprine, methotrexate, ciclosporin or mycophenolate).

Eczema severity
We then classified individuals as having: (1) mild eczema as default, (2) moderate disease from the first prescription of potent topical steroids, or calcineurin inhibitors; or (2) severe atopic eczema from the first referral to a dermatologist, use of phototherapy or prescribed systemic treatments.

Psoriasis
We identified individuals with psoriasis from the first date that they received a diagnostic code of psoriasis. We used the same 36 Read codes as a previous study of psoriasis [78].

Psoriasis severity
We defined individuals as exposed to mild psoriasis by default and then classified individuals as 'moderate/severe' from the first record of prescriptions for systemic therapies used to treat psoriasis [79]. We used two categories of psoriasis severity (as opposed to three levels for eczema severity) because of a lack of certainty of psoriasis severity phenotyping using primary care electronic health records. Relevant treatments that resulted in a "severe" psoriasis definition included: (1) systemic treatment (e.g., acitretin, etretinate, ciclosporin); (2) phototherapy; (3) biologic therapy (e.g., etanercept, adalimumab, infliximab).

Covariates
We defined 13 covariates in total in this study. These were categorised as follows and in line with directed acyclic graphs (Figure 1):

- Matching variables
  - Age
  - Sex
  - Practice
- Confounding variables
  - A specific comorbidity (asthma for eczema studies, psoriatic arthritis for psoriasis studies)
  - Charlson comorbidity index (CCI)
  - Index of deprivation
  - Ethnicity (sensitivity analysis only)
- Mediators
  - Harmful alcohol use
  - Body mass index (BMI)
  - Smoking status
  - Sleep problems (eczema studies only)
  - High dose oral glucocorticoids (eczema studies only)

These variables were defined as follows:

- Age, sex, and calendar period: all are available directly from CPRD.
- Deprivation: measured through the Carstairs index [46] from linked data to enable measurement across the UK. Quintiles of Carstairs index were used at the individual level where available, and practice level if not.
- Ethnicity: identified based on a previously validated algorithm using primary care morbidity coding [48].

- Comorbidity: we identified specific comorbidities for eczema (asthma) and psoriasis (psoriatic arthritis) based on morbidity coding in CPRD.
- Multimorbidity: to capture multiple chronic conditions we defined the Charlson comorbidity index (CCI). CCI assigns weights to each of the 17 conditions included in the index and is categorised as low (0), medium (1-2) or high (3+) [47]. We used the same morbidity codes for the definition of the 17 conditions in the definition of CCI as has been used in previous research [51]. Each condition in the CCI is weighted from one to six, with a weight of six representing the most severe morbidity.3 The sum of the weights in each individual results in a single comorbidity summary score. We categorised CCI scores into 3 groups: low (0 points), intermediate (1-2 points), and high (>=3 points).

The mediators included were:

- Harmful alcohol use: based on primary care records for morbidity codes suggesting harmful alcohol use or prescriptions for drugs used to maintain abstinence.
- Body mass index (BMI) measured using the height and weight recorded closest to the cohort entry date. BMI (kg m^{-2}) was grouped using categories derived from the WHO classification of BMI: no evidence of obesity, BMI < 30; obese class I, BMI 30–34.9; obese class II, BMI 35–39.9; and obese class III, BMI 40+. No record of BMI was assumed to indicate no evidence of obesity.
- Smoking status: Smoking status was classified as: (1) non-smoker; (2) current smoker; (3) ex-smoker; or (4) current/ex-smoker. Smoking status was time-updated however to reduce missing data we used the first observation carried back if data were missing at study entry.

In the eczema cohort we included two additional mediators:

- Sleep problems: based on: 1) primary care morbidity coding for sleep problems (e.g., insomnia); or 2) primary care prescribing of drugs used to manage sleep problems (e.g., zopiclone), specifically those prescribed at night.
- High dose oral glucocorticoids: based on primary care prescription records (20mg/day or more prednisolone equivalent dose) defined using primary care prescription records [2]. We defined a time updated 90-day risk window for high-dose glucocorticoids that began on the date of a prescription that met our high-dose criteria and continued for 90 days unless there was a subsequent prescription within that 90-day window (in which case the risk window continued from the date of the subsequent prescription) therefore individuals could have multiple 90-day risk windows during follow-up

Statistical analysis

Rate differences

We estimated incidence rate differences by first estimating the incidence rate of anxiety/depression in the exposed eczema/psoriasis population. We then multiplied this rate by the inverted hazard ratio from the confounder-adjusted Cox model to estimate the incidence rates of anxiety/depression among patients without

eczema/psoriasis, which cannot be reliably estimated from the sample owing to the matched study design. We repeated this process over 5,000 bootstrap samples to obtain uncertainty intervals, using a random draw of the log hazard ratio with the mean and standard deviation from the confounder-adjusted mode in each iteration.

Assessing proportional hazards & time-varying hazards
We assessed the proportional hazards (PH) assumption in the mediator-adjusted model as this was our primary finding. We assessed the PH assumption by plotting scaled Schoenfeld residuals over time. The smoothed spline through these residuals should then be equivalent to the estimated HR from the model and should be constant over time if the PH assumption is met. Where deviations from the PH were found we developed the same models incorporating time effects in two ways: (1) a linear interaction with time; (2) an interaction with time as a flexible parametric penalised spline.

We used the `*pspline*` function from the Survival package in R [53] to fit a Cox model with all confounders and mediators as included in our main analysis and an additional interaction between the exposure (eczema/psoriasis) and the penalised spline of time (days). The time-varying hazard ratio is then calculated as the linear combination of the coefficients for the exposure and the penalised spline. All analysis code is available online with the penalised spline modelling available here.

Sensitivity analysis
To assess robustness of our results to different assumptions we conducted two additional sensitivity analysis with all three models. First, we included ethnicity as an additional confounder, only in participants who joined the cohort from 2006 onwards for better data completeness for ethnicity. Secondly, we excluded individuals from the analysis that did not have at least one consultation with their GP in either 1-year or 3-years prior to study entry. These analyses removed individuals who never/rarely consult their practice, which may be more likely in the unexposed cohort. The reason for non-attendance at the practice may be because the record is incorrect and the individual no longer attends that practice, therefore making them appear artificially “healthy” in our analysis. However, the non-attendance could be due to genuine good health so we used these as sensitivity analyses instead of as the main analysis.

Computer software
Data management was performed in STATA version 16 [55] and R version 4.1.2 [56]. All analyses were performed in R version 4.1.2 and all code lists and analysis code are available online [57]. R packages were used for importing and manipulating data [58-61], and analysing data [53, 62-63].

Supplementary tables

Table 1: Full baseline characteristics of people with and without eczema at cohort entry for anxiety/depression. Data are N (%) if categorical, and median (IQR) if continuous.

Eczema	Anxiety cohort		Depression cohort	
Characteristic	Unexposed, N = 3,720,4781	Eczema, N = 863,9861	Unexposed, N = 3,228,8841	Eczema, N = 793,2961
**Follow-up time (years)**	4.5 (1.7-9.1)	4.9 (1.9-9.5)	4.4 (1.7-8.9)	4.7 (1.8-9.3)
**Age**	2,023,172 (54%)	484,720 (56%)	1,665,708 (52%)	431,807 (54%)
18-39	38.6 (23.8-59.6)	39.9 (24.1-60.8)	37.3 (22.8-59.6)	39.3 (23.4-61.0)
40-49	1,936,683 (52%)	432,946 (50%)	1,729,474 (54%)	404,386 (51%)
50-59	456,639 (12%)	107,595 (12%)	365,872 (11%)	93,802 (12%)
60-69	416,104 (11%)	99,094 (11%)	338,787 (10%)	87,368 (11%)
70-79	403,886 (11%)	96,209 (11%)	348,075 (11%)	88,132 (11%)
80+	317,043 (8.5%)	78,907 (9.1%)	283,161 (8.8%)	74,106 (9.3%)
**Sex (female)**	190,123 (5.1%)	49,235 (5.7%)	163,515 (5.1%)	45,502 (5.7%)
**Ethnicity**				
White	1,275,040 (84%)	313,460 (84%)	1,077,329 (84%)	282,827 (84%)
South Asian	77,386 (5.1%)	22,307 (6.0%)	69,095 (5.4%)	21,103 (6.3%)
Black	43,734 (2.9%)	9,921 (2.7%)	37,680 (2.9%)	9,033 (2.7%)
Other	34,577 (2.3%)	6,933 (1.9%)	30,187 (2.3%)	6,495 (1.9%)
Mixed	14,532 (1.0%)	3,433 (0.9%)	12,402 (1.0%)	3,107 (0.9%)
Not Stated	75,543 (5.0%)	15,894 (4.3%)	63,509 (4.9%)	14,307 (4.2%)
Unknown	2,199,666	492,038	1,938,682	456,424
**Carstairs deprivation quintile**				
1 (least deprived)	693,976 (19%)	164,020 (20%)	609,450 (20%)	153,083 (20%)
2	726,785 (20%)	169,436 (20%)	634,456 (20%)	156,814 (20%)
3	763,684 (21%)	177,947 (21%)	663,782 (21%)	163,294 (21%)
4	804,513 (22%)	185,506 (22%)	684,904 (22%)	167,523 (22%)
5 (most deprived)	616,149 (17%)	139,516 (17%)	529,446 (17%)	126,108 (16%)
Unknown	115,371	27,561	106,846	26,474
**Asthma diagnosis**	418,760 (11%)	194,298 (22%)	355,302 (11%)	175,497 (22%)
**Charlson's comorbidity index**				
Low	2,777,741 (75%)	542,649 (63%)	2,434,982 (75%)	503,973 (64%)
Moderate	803,045 (22%)	284,506 (33%)	681,188 (21%)	257,585 (32%)
Severe	139,692 (3.8%)	36,831 (4.3%)	112,714 (3.5%)	31,738 (4.0%)
**BMI**	25.2 (22.3-28.7)	25.4 (22.5-29.0)	25.1 (22.2-28.6)	25.2 (22.4-28.8)
Unknown	893,396	149,905	826,059	144,397
**Obesity (categorised)**				
no evidence of obesity	3,173,900 (85%)	717,101 (83%)	2,788,149 (86%)	667,059 (84%)
obese class I (30-34.9)	366,497 (9.9%)	96,216 (11%)	301,783 (9.3%)	84,553 (11%)
obese class II (35-39.9)	121,446 (3.3%)	33,420 (3.9%)	95,213 (2.9%)	28,048 (3.5%)
obese class III (40+)	58,635 (1.6%)	17,249 (2.0%)	43,739 (1.4%)	13,636 (1.7%)
**Harmful alcohol use**	96,328 (2.6%)	28,327 (3.3%)	72,753 (2.3%)	22,983 (2.9%)
**Smoking status**				
Non-Smoker	1,858,380 (57%)	456,720 (56%)	1,642,116 (58%)	431,014 (58%)
Current Smoker	642,990 (20%)	157,434 (19%)	523,575 (19%)	134,145 (18%)
Ex-Smoker	687,105 (21%)	181,117 (22%)	575,059 (20%)	163,142 (22%)
Current Or Ex-Smoker	97,395 (3.0%)	20,466 (2.5%)	76,982 (2.7%)	16,700 (2.2%)
Unknown	434,608	48,249	411,152	48,295
**Sleep problems**	257,752 (6.9%)	109,378 (13%)	190,349 (5.9%)	88,728 (11%)
**Severe eczema**				
None	3,720,478 (100%)	-	3,228,884 (100%)	-
Mild	-	638,977 (74%)	-	587,271 (74%)
Moderate	-	208,757 (24%)	-	190,798 (24%)
Severe	-	16,252 (1.9%)	-	15,227 (1.9%)

1 Median (IQR) or Frequency (%)

Table 2: Full baseline characteristics of people with and without psoriasis at cohort entry for anxiety/depression. Data are N (%) if categorical, and median (IQR) if continuous.

**Psoriasis**	**Anxiety cohort**		**Depression cohort**	
**Characteristic**	**Unexposed, N = 1,365,2581**	**Psoriasis, N = 310,0811**	**Unexposed, N = 1,177,2991**	**Psoriasis, N = 282,8661**
**Follow-up time (years)**	5.7 (2.3-11.0)	5.4 (2.1-10.9)	5.6 (2.2-10.9)	5.3 (2.1-10.6)
**Sex (Female)**	659,460 (48%)	155,059 (50%)	535,278 (45%)	136,554 (48%)
**Age**	43.4 (30.2-59.7)	44.0 (30.7-60.1)	43.0 (29.6-60.0)	43.8 (30.4-60.3)
18-39	603,064 (44%)	133,399 (43%)	529,725 (45%)	123,152 (44%)
40-49	223,800 (16%)	51,711 (17%)	184,885 (16%)	45,724 (16%)
50-59	201,742 (15%)	47,179 (15%)	168,318 (14%)	42,214 (15%)
60-69	168,935 (12%)	39,295 (13%)	146,033 (12%)	35,972 (13%)
70-79	114,154 (8.4%)	26,201 (8.4%)	101,858 (8.7%)	24,465 (8.6%)
80+	53,563 (3.9%)	12,296 (4.0%)	46,480 (3.9%)	11,339 (4.0%)
**Ethnicity**				
White	437,548 (87%)	124,555 (90%)	368,399 (87%)	111,683 (90%)
South Asian	19,435 (3.9%)	4,370 (3.2%)	17,035 (4.0%)	4,119 (3.3%)
Black	10,645 (2.1%)	972 (0.7%)	9,006 (2.1%)	852 (0.7%)
Other	8,857 (1.8%)	1,654 (1.2%)	7,628 (1.8%)	1,537 (1.2%)
Mixed	3,308 (0.7%)	711 (0.5%)	2,817 (0.7%)	643 (0.5%)
Not Stated	24,662 (4.9%)	6,214 (4.5%)	20,565 (4.8%)	5,588 (4.5%)
Unknown	860,803	171,605	751,849	158,444
**Carstairs deprivation quintile**				
1 (least deprived)	239,381 (18%)	54,491 (18%)	209,265 (19%)	50,577 (19%)
2	258,876 (20%)	58,095 (20%)	224,322 (20%)	53,369 (20%)
3	285,488 (22%)	64,962 (22%)	248,284 (22%)	59,633 (22%)
4	301,611 (23%)	68,634 (23%)	255,341 (23%)	61,631 (23%)
5 (most deprived)	222,091 (17%)	50,356 (17%)	187,961 (17%)	44,999 (17%)
Unknown	57,811	13,543	52,126	12,657
**Arthritis diagnosis**	8,572 (0.6%)	3,638 (1.2%)	6,841 (0.6%)	3,114 (1.1%)
**Charlson's comorbidity index**				
Low	1,010,496 (74%)	219,105 (71%)	881,218 (75%)	202,700 (72%)
Moderate	305,143 (22%)	77,690 (25%)	256,072 (22%)	68,853 (24%)
Severe	49,619 (3.6%)	13,286 (4.3%)	40,009 (3.4%)	11,313 (4.0%)
**BMI**	25.6 (22.7-29.1)	26.1 (23.1-29.8)	25.5 (22.7-28.9)	26.0 (23.1-29.7)
Unknown	272,289	45,254	248,099	42,476
**Obesity (categorised)**				
no evidence of obesity	1,141,895 (84%)	245,756 (79%)	996,546 (85%)	226,971 (80%)
obese class I (30-34.9)	151,030 (11%)	41,773 (13%)	125,007 (11%)	37,148 (13%)
obese class II (35-39.9)	49,001 (3.6%)	14,862 (4.8%)	38,407 (3.3%)	12,628 (4.5%)
obese class III (40+)	23,332 (1.7%)	7,690 (2.5%)	17,339 (1.5%)	6,119 (2.2%)
**Harmful alcohol use**	41,845 (3.1%)	13,901 (4.5%)	31,626 (2.7%)	11,147 (3.9%)
**Smoking status**				
Non-Smoker	662,851 (54%)	131,125 (45%)	580,837 (55%)	123,149 (46%)
Current Smoker	255,508 (21%)	77,270 (26%)	210,144 (20%)	66,833 (25%)
Ex-Smoker	273,966 (22%)	74,257 (25%)	229,228 (22%)	67,047 (25%)
Current Or Ex-Smoker	36,229 (2.9%)	9,614 (3.3%)	28,182 (2.7%)	7,951 (3.0%)
Unknown	136,704	17,815	128,908	17,886
**Sleep problems**	100,413 (7.4%)	27,945 (9.0%)	72,233 (6.1%)	21,366 (7.6%)
**Severe psoriasis**				
None	1,365,258 (100%)	0 (0%)	1,177,299 (100%)	0 (0%)
Mild	0 (0%)	303,327 (98%)	0 (0%)	277,113 (98%)
Severe	0 (0%)	6,754 (2.2%)	0 (0%)	5,753 (2.0%)

1: Median (IQR) or Frequency (%)

Table 3: Data availability for quintiles of the Carstairs index of deprivation. Patient-level data was used where available, practice-level if not. Data for all practices were not available from record linkage.

	Missing	%	Recorded	%
Practice level				
Unexposed (eczema cohort)	269,386	5.4	4,720,739	94.6
Eczema	55,574	5.4	977,208	94.6
Unexposed (psoriasis cohort)	99,948	5.4	1,734,382	94.6
Psoriasis	19,990	5.4	346,894	94.6
Patient level				
Unexposed (eczema cohort)	2,571,483	51.5	2,418,642	48.5
Eczema	492,046	47.6	540,736	52.4
Unexposed (psoriasis cohort)	1,044,130	56.9	790,200	43.1
Psoriasis	188,564	51.4	178,320	48.6

Table 4: Characteristics of time updated covariates throughout the entire study period (1997-2020). This table shows the number (%) of participants with a record at any point during their eligible follow up

	Anxiety cohort		Depression cohort	
**Characteristic**	**Unexposed**	**Exposed**	**Unexposed**	**Exposed**
**Eczema cohort (n)**	3,720,478	863,986	3,228,884	793,296
**Harmful alcohol use**	180,397 (4.8%)	51,480 (6.0%)	138,948 (4.3%)	42,381 (5.3%)
**Smoking status**				
Non-Smoker	1,858,380 (57%)	456,720 (56%)	1,642,116 (58%)	431,014 (58%)
Ex-Smoker	687,105 (21%)	181,117 (22%)	575,059 (20%)	163,142 (22%)
Current Or Ex-Smoker	97,395 (3.0%)	20,466 (2.5%)	76,982 (2.7%)	16,700 (2.2%)
Current Smoker	642,990 (20%)	157,434 (19%)	523,575 (19%)	134,145 (18%)
Unknown	434,608	48,249	411,152	48,295
**Sleep problems**	464,583 (12%)	178,254 (21%)	346,131 (11%)	144,623 (18%)
**Asthma diagnosis**	484,529 (13%)	217,044 (25%)	408,432 (13%)	195,095 (25%)
**Severe eczema**				
None	3,688,936 (99%)	0 (0%)	3,202,440 (99%)	0 (0%)
Mild	0 (0%)	522,136 (60%)	0 (0%)	482,500 (61%)
Moderate	29,167 (0.8%)	316,448 (37%)	24,582 (0.8%)	287,205 (36%)
Severe	2,375 (<0.1%)	25,402 (2.9%)	1,862 (<0.1%)	23,591 (3.0%)
**High dose oral steroid prescription**	358,092 (9.6%)	154,810 (18%)	288,269 (8.9%)	133,574 (17%)
**Psoriasis cohort (n)**	1,365,258	310,081	1,177,299	282,866
**Harmful alcohol use**	86,066 (6.3%)	25,971 (8.4%)	67,441 (5.7%)	21,352 (7.5%)
**Smoking status**				
Non-Smoker	662,851 (54%)	131,125 (45%)	580,837 (55%)	123,149 (46%)
Ex-Smoker	273,966 (22%)	74,257 (25%)	229,228 (22%)	67,047 (25%)
Current Or Ex-Smoker	36,229 (2.9%)	9,614 (3.3%)	28,182 (2.7%)	7,951 (3.0%)
Current Smoker	255,508 (21%)	77,270 (26%)	210,144 (20%)	66,833 (25%)
Unknown	136,704	17,815	128,908	17,886
**Arthritis diagnosis**	13,264 (1.0%)	5,697 (1.8%)	10,684 (0.9%)	4,904 (1.7%)
**Severe psoriasis**				
None	1,365,026 (100%)	0 (0%)	1,177,119 (100%)	0 (0%)
Mild	0 (0%)	292,487 (94%)	0 (0%)	267,976 (95%)
Moderate	0 (0%)	0 (0%)	0 (0%)	0 (0%)
Severe	232 (<0.1%)	17,594 (5.7%)	180 (<0.1%)	14,890 (5.3%)

[1] Median (IQR) or Frequency (%)

*Table 5: Person years under follow up in adults **with and without eczema** by individual-level characteristics. Values are person-years at risk (%)*

	Eczema cohort			
	Anxiety cohort		Depression cohort	
**Characteristic**	**Matched controls**	**With eczema**	**Matched controls**	**With eczema**
**Sex**				
Male	3,820,032,627.2 (46.8%)	881,513,844.0 (44.3%)	3,492,834,747.8 (50.1%)	828,536,917.5 (46.4%)
Female	4,339,935,742.8 (53.2%)	1,106,887,896.0 (55.7%)	3,481,803,212.8 (49.9%)	955,367,179.2 (53.6%)
**Age (categorised)**				
18-39	3,016,715,004.2 (37.0%)	713,231,629.8 (35.9%)	2,653,179,576.2 (38.0%)	648,043,633.0 (36.3%)
40-49	1,267,220,075.8 (15.5%)	306,660,040.8 (15.4%)	1,013,420,384.8 (14.5%)	262,155,532.0 (14.7%)
50-59	1,185,091,999.2 (14.5%)	290,040,025.0 (14.6%)	963,211,845.8 (13.8%)	250,839,110.8 (14.1%)
60-69	1,123,030,304.8 (13.8%)	276,578,625.5 (13.9%)	951,238,129.2 (13.6%)	248,292,081.2 (13.9%)
70-79	946,997,494.5 (11.6%)	238,681,225.2 (12.0%)	845,263,598.2 (12.1%)	223,029,045.8 (12.5%)
80+	620,913,491.5 (7.6%)	163,210,193.8 (8.2%)	548,324,426.2 (7.9%)	151,544,694.0 (8.5%)
**Asthma diagnosis**				
No	7,184,421,454.2 (88.0%)	1,520,204,549.5 (76.5%)	6,171,752,846.2 (88.5%)	1,376,571,060.2 (77.2%)
Yes	975,546,915.8 (12.0%)	468,197,190.5 (23.5%)	802,885,114.2 (11.5%)	407,333,036.5 (22.8%)
**Calendar period**				
1997-2002	920,064,579.0 (11.3%)	210,019,745.5 (10.6%)	814,659,863.0 (11.7%)	193,837,107.2 (10.9%)
2002-08	2,145,222,160.5 (26.3%)	510,215,087.2 (25.7%)	1,864,632,390.0 (26.7%)	462,675,016.5 (25.9%)
2008-14	2,934,102,211.0 (36.0%)	725,741,459.8 (36.5%)	2,487,025,679.2 (35.7%)	646,854,771.8 (36.3%)
2014-19	2,160,579,419.5 (26.5%)	542,425,447.5 (27.3%)	1,808,320,028.2 (25.9%)	480,537,201.2 (26.9%)
**Carstairs deprivation quintile**				
1 (Least Deprived)	1,572,765,649.2 (19.3%)	385,772,151.0 (19.4%)	1,368,188,145.2 (19.6%)	354,158,662.0 (19.9%)
2	1,583,379,109.2 (19.4%)	384,905,123.2 (19.4%)	1,365,216,782.0 (19.6%)	349,264,793.2 (19.6%)
3	1,663,186,724.8 (20.4%)	405,596,195.8 (20.4%)	1,423,230,980.8 (20.4%)	363,259,035.0 (20.4%)
4	1,737,772,595.0 (21.3%)	422,073,057.8 (21.2%)	1,448,135,804.2 (20.8%)	369,724,296.8 (20.7%)
5 (Most Deprived)	1,287,534,092.8 (15.8%)	311,728,628.2 (15.7%)	1,079,199,156.0 (15.5%)	273,087,750.5 (15.3%)
NA	315,330,199.0 (3.9%)	78,326,584.0 (3.9%)	290,667,092.2 (4.2%)	74,409,559.2 (4.2%)
**Charlson's comorbidity index**				
Low	6,283,063,029.8 (77.0%)	1,299,436,596.2 (65.4%)	5,415,173,475.2 (77.6%)	1,179,806,248.5 (66.1%)
Moderate	1,664,917,590.0 (20.4%)	626,487,879.0 (31.5%)	1,389,032,945.8 (19.9%)	551,120,464.5 (30.9%)
Severe	211,987,750.2 (2.6%)	62,477,264.8 (3.1%)	170,431,539.5 (2.4%)	52,977,383.8 (3.0%)
**Ethnicity**				
White	2,802,664,534.5 (34.3%)	731,608,923.2 (36.8%)	2,343,758,014.2 (33.6%)	647,681,454.2 (36.3%)

South Asian	114,639,509.5 (1.4%)	40,785,865.2 (2.1%)	101,013,640.0 (1.4%)	37,762,756.0 (2.1%)
Black	61,660,306.8 (0.8%)	16,668,203.0 (0.8%)	51,668,756.5 (0.7%)	14,602,109.0 (0.8%)
Other	45,214,355.8 (0.6%)	12,080,094.2 (0.6%)	38,789,300.8 (0.6%)	11,167,839.5 (0.6%)
Mixed	19,140,700.2 (0.2%)	5,296,008.8 (0.3%)	15,910,403.8 (0.2%)	4,718,302.0 (0.3%)
Not Stated	140,382,503.2 (1.7%)	31,875,199.5 (1.6%)	115,231,239.5 (1.7%)	27,890,737.2 (1.6%)
NA	4,976,266,460.0 (61.0%)	1,150,087,446.0 (57.8%)	4,308,266,605.8 (61.8%)	1,040,080,898.8 (58.3%)
**Harmful alcohol use**				
No	7,867,109,224.0 (96.4%)	1,902,765,180.0 (95.7%)	6,751,131,456.2 (96.8%)	1,714,797,509.5 (96.1%)
Yes	292,859,146.0 (3.6%)	85,636,560.0 (4.3%)	223,506,504.2 (3.2%)	69,106,587.2 (3.9%)
**Obesity (categorised)**				
No Evidence Of Obesity	6,867,418,784.0 (84.2%)	1,631,296,126.8 (82.0%)	5,944,888,124.2 (85.2%)	1,486,200,739.5 (83.3%)
Obese Class I (30-34.9)	883,591,125.2 (10.8%)	238,529,536.8 (12.0%)	719,884,828.0 (10.3%)	203,940,941.8 (11.4%)
Obese Class Ii (35-39.9)	280,558,432.2 (3.4%)	79,317,545.5 (4.0%)	216,489,896.2 (3.1%)	64,027,480.5 (3.6%)
Obese Class Iii (40+)	128,400,028.5 (1.6%)	39,258,531.0 (2.0%)	93,375,112.0 (1.3%)	29,734,935.0 (1.7%)
**Rural/Urban**				
Urban	7,063,673,686.2 (86.6%)	1,723,606,429.2 (86.7%)	6,028,427,745.2 (86.4%)	1,543,274,365.8 (86.5%)
Rural	1,096,294,683.8 (13.4%)	264,795,310.8 (13.3%)	946,210,215.2 (13.6%)	240,629,731.0 (13.5%)
**Severity**				
None	8,159,968,370.0 (100.0%)	NA (NA%)	6,974,637,960.5 (100.0%)	NA (NA%)
Mild	NA (NA%)	1,204,749,656.5 (60.6%)	NA (NA%)	1,085,596,443.2 (60.9%)
Moderate	NA (NA%)	721,429,093.2 (36.3%)	NA (NA%)	642,152,459.8 (36.0%)
Severe	NA (NA%)	62,222,990.2 (3.1%)	NA (NA%)	56,155,193.8 (3.1%)
**Sleep problems**				
No	7,330,629,741.5 (89.8%)	1,646,963,594.5 (82.8%)	6,379,233,610.2 (91.5%)	1,517,151,829.5 (85.0%)
Yes	829,338,628.5 (10.2%)	341,438,145.5 (17.2%)	595,404,350.2 (8.5%)	266,752,267.2 (15.0%)
**Smoking status**				
Non-Smoker	4,352,350,365.8 (53.3%)	1,107,115,583.0 (55.7%)	3,797,101,234.8 (54.4%)	1,024,551,899.5 (57.4%)
Current Smoker	1,572,306,618.2 (19.3%)	394,795,916.5 (19.9%)	1,257,048,409.0 (18.0%)	327,769,263.2 (18.4%)
Ex-Smoker	1,458,094,722.5 (17.9%)	398,940,144.0 (20.1%)	1,218,854,041.5 (17.5%)	353,427,923.8 (19.8%)
Current Or Ex-Smoker	170,697,761.5 (2.1%)	39,743,662.8 (2.0%)	132,804,265.0 (1.9%)	31,451,184.8 (1.8%)
NA	606,518,902.0 (7.4%)	47,806,433.8 (2.4%)	568,830,010.2 (8.2%)	46,703,825.5 (2.6%)

*Table 6: Person years under follow up in adults **with and without psoriasis** by individual-level characteristics. Values are person-years at risk (%)*

	Psoriasis cohort			
	Anxiety cohort		Depression cohort	
**Characteristic**	**Matched controls**	**With psoriasis**	**Matched controls**	**With psoriasis**
**Sex**				
Male	1,871,928,693.0 (52.9%)	406,966,938.5 (51.4%)	1,698,575,005.8 (56.0%)	379,170,956.0 (53.5%)
Female	1,668,731,007.0 (47.1%)	384,788,400.8 (48.6%)	1,333,623,628.0 (44.0%)	329,339,291.5 (46.5%)
**Age (categorised)**				
18-39	1,012,272,241.5 (28.6%)	218,559,204.0 (27.6%)	876,636,946.0 (28.9%)	196,301,131.2 (27.7%)
40-49	659,993,866.8 (18.6%)	148,603,698.8 (18.8%)	546,291,186.5 (18.0%)	129,648,822.8 (18.3%)
50-59	643,415,104.2 (18.2%)	146,415,023.0 (18.5%)	538,793,082.0 (17.8%)	128,637,211.8 (18.2%)
60-69	572,735,561.0 (16.2%)	131,764,863.2 (16.6%)	491,917,156.0 (16.2%)	118,724,943.2 (16.8%)
70-79	423,547,889.8 (12.0%)	96,437,069.8 (12.2%)	376,605,929.0 (12.4%)	89,304,189.0 (12.6%)
80+	228,695,036.8 (6.5%)	49,975,480.5 (6.3%)	201,954,334.2 (6.7%)	45,893,949.5 (6.5%)
**Arthritis diagnosis**				
No	3,510,197,737.8 (99.1%)	778,709,199.0 (98.4%)	3,007,978,978.5 (99.2%)	697,539,618.0 (98.5%)
Yes	30,461,962.2 (0.9%)	13,046,140.2 (1.6%)	24,219,655.2 (0.8%)	10,970,629.5 (1.5%)
**Calendar period**				
1997-2002	391,228,028.0 (11.0%)	87,273,979.5 (11.0%)	351,509,651.2 (11.6%)	81,422,901.2 (11.5%)
2002-08	989,031,583.0 (27.9%)	221,769,212.2 (28.0%)	870,584,108.5 (28.7%)	202,634,892.0 (28.6%)
2008-14	1,251,518,597.0 (35.3%)	280,338,910.2 (35.4%)	1,061,036,942.0 (35.0%)	248,531,871.8 (35.1%)
2014-19	908,881,492.0 (25.7%)	202,373,237.2 (25.6%)	749,067,932.0 (24.7%)	175,920,582.5 (24.8%)
**Carstairs deprivation quintile**				
1 (Least Deprived)	641,425,623.8 (18.1%)	142,411,153.5 (18.0%)	560,193,154.0 (18.5%)	130,954,574.0 (18.5%)
2	653,185,080.2 (18.4%)	144,121,637.8 (18.2%)	562,778,204.8 (18.6%)	130,124,782.8 (18.4%)
3	738,976,449.2 (20.9%)	165,058,183.2 (20.8%)	637,968,250.2 (21.0%)	148,956,745.2 (21.0%)
4	769,789,411.5 (21.7%)	172,976,765.2 (21.8%)	643,580,508.8 (21.2%)	151,754,053.0 (21.4%)
5 (Most Deprived)	558,459,117.8 (15.8%)	125,007,186.2 (15.8%)	467,588,901.0 (15.4%)	108,500,936.5 (15.3%)
NA	178,824,017.5 (5.1%)	42,180,413.2 (5.3%)	160,089,615.0 (5.3%)	38,219,156.0 (5.4%)
**Charlson's comorbidity index**				
Low	2,719,569,952.5 (76.8%)	586,166,340.8 (74.0%)	2,353,348,921.2 (77.6%)	531,513,378.8 (75.0%)
Moderate	736,835,153.0 (20.8%)	183,098,688.8 (23.1%)	611,286,014.5 (20.2%)	158,219,187.5 (22.3%)

Severe	84,254,594.5 (2.4%)	22,490,309.8 (2.8%)	67,563,698.0 (2.2%)	18,777,681.2 (2.7%)
**Ethnicity**				
White	1,196,228,316.8 (33.8%)	313,098,302.0 (39.5%)	1,009,886,809.2 (33.3%)	276,528,598.5 (39.0%)
South Asian	35,103,021.8 (1.0%)	7,818,095.2 (1.0%)	30,153,157.2 (1.0%)	7,158,059.2 (1.0%)
Black	17,753,061.8 (0.5%)	1,679,952.5 (0.2%)	14,701,108.5 (0.5%)	1,420,208.2 (0.2%)
Other	15,232,395.5 (0.4%)	2,934,383.5 (0.4%)	13,044,062.2 (0.4%)	2,718,252.5 (0.4%)
Mixed	5,416,327.8 (0.2%)	1,137,844.8 (0.1%)	4,420,371.8 (0.1%)	987,654.5 (0.1%)
Not Stated	59,168,932.8 (1.7%)	14,288,665.0 (1.8%)	49,165,064.8 (1.6%)	12,617,098.2 (1.8%)
NA	2,211,757,643.8 (62.5%)	450,798,096.2 (56.9%)	1,910,828,060.0 (63.0%)	407,080,376.2 (57.5%)
**Harmful alcohol use**				
No	3,381,190,574.0 (95.5%)	744,422,962.0 (94.0%)	2,907,505,327.5 (95.9%)	670,065,697.0 (94.6%)
Yes	159,469,126.0 (4.5%)	47,332,377.2 (6.0%)	124,693,306.2 (4.1%)	38,444,550.5 (5.4%)
**Obesity (categorised)**				
No Evidence Of Obesity	2,933,780,996.8 (82.9%)	623,946,751.0 (78.8%)	2,543,388,092.0 (83.9%)	566,125,831.0 (79.9%)
Obese Class I (30-34.9)	418,660,457.8 (11.8%)	112,032,787.0 (14.1%)	344,945,611.0 (11.4%)	97,474,596.8 (13.8%)
Obese Class Ii (35-39.9)	129,694,590.8 (3.7%)	37,545,890.0 (4.7%)	101,175,459.2 (3.3%)	30,899,672.5 (4.4%)
Obese Class Iii (40+)	58,523,654.8 (1.7%)	18,229,911.2 (2.3%)	42,689,471.5 (1.4%)	14,010,147.2 (2.0%)
**Rural/Urban**				
Urban	3,067,584,987.5 (86.6%)	687,528,179.2 (86.8%)	2,620,008,291.5 (86.4%)	613,769,065.5 (86.6%)
Rural	473,074,712.5 (13.4%)	104,227,160.0 (13.2%)	412,190,342.2 (13.6%)	94,741,182.0 (13.4%)
**Severity**				
None	3,540,659,700.0 (100.0%)	NA (NA%)	3,032,198,633.8 (100.0%)	NA (NA%)
Mild	NA (NA%)	752,619,516.5 (95.1%)	NA (NA%)	676,121,658.0 (95.4%)
Severe	NA (NA%)	39,135,822.8 (4.9%)	NA (NA%)	32,388,589.5 (4.6%)
**Smoking status**				
Non-Smoker	1,852,683,401.0 (52.3%)	361,097,184.5 (45.6%)	1,617,494,548.0 (53.3%)	332,766,465.5 (47.0%)
Current Smoker	732,603,544.5 (20.7%)	211,952,536.8 (26.8%)	596,082,281.8 (19.7%)	179,972,170.2 (25.4%)
Ex-Smoker	668,735,841.5 (18.9%)	178,705,575.5 (22.6%)	562,257,043.8 (18.5%)	159,885,551.2 (22.6%)
Current Or Ex-Smoker	71,577,182.8 (2.0%)	19,292,294.8 (2.4%)	55,478,405.0 (1.8%)	15,473,452.5 (2.2%)
NA	215,059,730.2 (6.1%)	20,707,747.8 (2.6%)	200,886,355.2 (6.6%)	20,412,608.0 (2.9%)

Table 7: Baseline characteristics of those with and without complete data, comparing adults ***with and without eczema****. Value are number of individuals (%). Variable with missingness are Carstairs index of deprivation, BMI, Smoking status*

	Unexposed			Eczema		
**Characteristic**	**Complete, N = 1,391,0261**	**Missing other data, N = 1,220,1531**	**Missing ethnicity data, N = 1,528,7511**	**Complete, N = 346,7201**	**Missing other data, N = 209,5381**	**Missing ethnicity data, N = 376,1061**
**Age at index**	42.4 (28.1-60.6)	26.0 (18.1-45.7)	46.4 (29.7-64.6)	43.0 (28.9-60.8)	23.1 (18.1-42.5)	46.8 (29.6-65.6)
**Sex**						
Male	552,140 (40%)	671,084 (55%)	636,171 (42%)	136,841 (39%)	115,278 (55%)	151,353 (40%)
Female	838,886 (60%)	549,069 (45%)	892,580 (58%)	209,879 (61%)	94,260 (45%)	224,753 (60%)
**Ethnicity**						
White	1,181,355 (85%)	251,068 (81%)	0 (NA%)	294,417 (85%)	46,324 (83%)	0 (NA%)
South Asian	69,341 (5.0%)	15,404 (5.0%)	0 (NA%)	20,702 (6.0%)	2,863 (5.1%)	0 (NA%)
Black	38,004 (2.7%)	9,473 (3.0%)	0 (NA%)	8,647 (2.5%)	1,678 (3.0%)	0 (NA%)
Other	28,516 (2.0%)	9,213 (3.0%)	0 (NA%)	6,187 (1.8%)	1,077 (1.9%)	0 (NA%)
Mixed	12,011 (0.9%)	3,975 (1.3%)	0 (NA%)	2,985 (0.9%)	675 (1.2%)	0 (NA%)
Not Stated	61,799 (4.4%)	21,714 (7.0%)	0 (NA%)	13,782 (4.0%)	3,264 (5.8%)	0 (NA%)
equally common	0 (0%)	0 (0%)	0 (NA%)	0 (0%)	0 (0%)	0 (NA%)
Unknown	0	909,306	1,528,751	0	153,657	376,106
**Carstairs index of deprivation**						
1 (least deprived)	256,050 (18%)	203,723 (19%)	311,736 (20%)	64,073 (18%)	33,817 (19%)	78,609 (21%)
2	276,891 (20%)	214,328 (20%)	317,114 (21%)	68,500 (20%)	36,389 (20%)	77,845 (21%)
3	294,546 (21%)	226,983 (21%)	328,759 (22%)	74,599 (22%)	37,906 (21%)	79,453 (21%)
4	306,692 (22%)	242,430 (22%)	343,144 (22%)	75,795 (22%)	39,842 (22%)	84,346 (22%)
5 (most deprived)	256,847 (18%)	199,696 (18%)	227,998 (15%)	63,753 (18%)	31,029 (17%)	55,853 (15%)
Unknown	0	132,993	0	0	30,555	0
**Calendar Period**						
1997-2001	196,939 (14%)	317,403 (26%)	530,755 (35%)	49,495 (14%)	53,280 (25%)	123,437 (33%)
2002-07	300,213 (22%)	316,986 (26%)	548,452 (36%)	78,858 (23%)	50,297 (24%)	129,016 (34%)
2008-13	574,806 (41%)	331,209 (27%)	301,377 (20%)	138,253 (40%)	56,885 (27%)	81,544 (22%)
2014-19	319,068 (23%)	254,555 (21%)	148,167 (9.7%)	80,114 (23%)	49,076 (23%)	42,109 (11%)
**Psoriatic Arthritis**	169,631 (12%)	121,510 (10.0%)	176,546 (12%)	81,367 (23%)	48,244 (23%)	80,160 (21%)
**Charlson comorbidity index**						
Low (0)	997,566 (72%)	996,188 (82%)	1,087,982 (71%)	213,007 (61%)	140,193 (67%)	231,800 (62%)
Moderate (1-2)	330,066 (24%)	200,615 (16%)	369,966 (24%)	117,928 (34%)	63,753 (30%)	125,635 (33%)
Severe (3+)	63,394 (4.6%)	23,350 (1.9%)	70,803 (4.6%)	15,785 (4.6%)	5,592 (2.7%)	18,671 (5.0%)
**Obesity status**						

no evidence of obesity	1,103,839 (79%)	1,179,594 (97%)	1,244,540 (81%)	270,845 (78%)	200,365 (96%)	302,665 (80%)
obese class I (30-34.9)	189,944 (14%)	27,346 (2.2%)	193,267 (13%)	49,003 (14%)	6,116 (2.9%)	48,749 (13%)
obese class II (35-39.9)	65,087 (4.7%)	8,826 (0.7%)	62,006 (4.1%)	17,689 (5.1%)	2,001 (1.0%)	16,390 (4.4%)
obese class III (40+)	32,156 (2.3%)	4,387 (0.4%)	28,938 (1.9%)	9,183 (2.6%)	1,056 (0.5%)	8,302 (2.2%)
**Body Mass Index (BMI)**	25.4 (22.4-29.1)	24.5 (21.6-28.0)	25.1 (22.3-28.6)	25.6 (22.6-29.3)	24.7 (21.9-28.4)	25.2 (22.4-28.8)
Unknown	0	973,300	0	0	158,652	0
**Harmful alcohol use**	56,531 (4.1%)	15,867 (1.3%)	37,031 (2.4%)	16,678 (4.8%)	3,678 (1.8%)	11,079 (2.9%)
**Smoking status**						
Non-Smoker	737,709 (53%)	458,001 (63%)	864,935 (57%)	180,098 (52%)	98,337 (64%)	210,479 (56%)
Current Smoker	253,412 (18%)	155,064 (21%)	303,234 (20%)	64,449 (19%)	30,717 (20%)	74,589 (20%)
Ex-Smoker	352,303 (25%)	93,388 (13%)	325,485 (21%)	92,007 (27%)	21,016 (14%)	83,246 (22%)
Current Or Ex-Smoker	47,602 (3.4%)	25,629 (3.5%)	35,097 (2.3%)	10,166 (2.9%)	4,236 (2.7%)	7,792 (2.1%)
Unknown	0	488,071	0	0	55,232	0
**Sleep problems**	108,404 (7.8%)	69,695 (5.7%)	119,740 (7.8%)	46,704 (13%)	26,080 (12%)	49,559 (13%)
**High dose oral gluccocorticoid use**	12,942 (0.9%)	4,951 (0.4%)	16,361 (1.1%)	12,998 (3.7%)	4,334 (2.1%)	14,703 (3.9%)

[1] Median (IQR) or Frequency (%)

*Table 8: Baseline characteristics of those with and without complete data, comparing adults **with and without psoriasis**. Value are number of individuals (%). Variable with missingness are Carstairs index of deprivation, BMI, Smoking status*

	Unexposed			Psoriasis		
**Characteristic**	**Complete, N = 485,1571**	**Missing other data, N = 397,8541**	**Missing ethnicity data, N = 631,0671**	**Complete, N = 129,4061**	**Missing other data, N = 72,8691**	**Missing ethnicity data, N = 130,7421**
**Age at index**	46.5 (33.6-60.9)	34.0 (23.1-50.5)	47.6 (33.5-62.8)	45.6 (33.1-60.0)	34.5 (23.1-51.4)	48.0 (33.6-63.1)
**Sex**						
Male	228,741 (47%)	240,028 (60%)	302,867 (48%)	61,246 (47%)	41,007 (56%)	62,150 (48%)
Female	256,416 (53%)	157,826 (40%)	328,200 (52%)	68,160 (53%)	31,862 (44%)	68,592 (52%)
**Ethnicity**						
White	424,127 (87%)	66,516 (84%)	0 (NA%)	116,841 (90%)	17,682 (89%)	0 (NA%)
South Asian	18,344 (3.8%)	2,977 (3.8%)	0 (NA%)	4,075 (3.1%)	501 (2.5%)	0 (NA%)
Black	9,914 (2.0%)	1,691 (2.1%)	0 (NA%)	878 (0.7%)	130 (0.7%)	0 (NA%)
Other	7,898 (1.6%)	1,818 (2.3%)	0 (NA%)	1,473 (1.1%)	253 (1.3%)	0 (NA%)
Mixed	3,064 (0.6%)	630 (0.8%)	0 (NA%)	655 (0.5%)	104 (0.5%)	0 (NA%)
Not Stated	21,810 (4.5%)	5,465 (6.9%)	0 (NA%)	5,484 (4.2%)	1,177 (5.9%)	0 (NA%)
equally common	0 (0%)	0 (0%)	0 (NA%)	0 (0%)	0 (0%)	0 (NA%)
Unknown	0	318,757	631,067	0	53,022	130,742

**Carstairs index of deprivation**						
1 (least deprived)	83,711 (17%)	57,155 (17%)	123,942 (20%)	23,074 (18%)	10,232 (18%)	24,936 (19%)
2	96,328 (20%)	63,956 (19%)	126,163 (20%)	25,233 (19%)	11,124 (19%)	25,859 (20%)
3	105,510 (22%)	71,835 (22%)	139,741 (22%)	27,953 (22%)	12,771 (22%)	29,139 (22%)
4	108,430 (22%)	77,154 (23%)	147,806 (23%)	29,228 (23%)	13,522 (23%)	30,860 (24%)
5 (most deprived)	91,178 (19%)	61,796 (19%)	93,415 (15%)	23,918 (18%)	10,359 (18%)	19,948 (15%)
Unknown	0	65,958	0	0	14,861	0
**Calendar Period**						
1997-2001	91,941 (19%)	124,767 (31%)	222,517 (35%)	21,225 (16%)	23,927 (33%)	49,036 (38%)
2002-07	123,538 (25%)	118,541 (30%)	218,983 (35%)	31,317 (24%)	20,587 (28%)	48,081 (37%)
2008-13	164,350 (34%)	89,659 (23%)	127,763 (20%)	49,369 (38%)	15,603 (21%)	20,288 (16%)
2014-19	105,328 (22%)	64,887 (16%)	61,804 (9.8%)	27,495 (21%)	12,752 (17%)	13,337 (10%)
**Psoriatic arthritis**	3,499 (0.7%)	1,395 (0.4%)	4,706 (0.7%)	1,608 (1.2%)	629 (0.9%)	1,664 (1.3%)
**Charlson comorbidity index**						
Low (0)	341,363 (70%)	325,999 (82%)	449,389 (71%)	88,742 (69%)	55,533 (76%)	90,395 (69%)
Moderate (1-2)	121,776 (25%)	64,947 (16%)	154,571 (24%)	34,488 (27%)	15,161 (21%)	34,281 (26%)
Severe (3+)	22,018 (4.5%)	6,908 (1.7%)	27,107 (4.3%)	6,176 (4.8%)	2,175 (3.0%)	6,066 (4.6%)
**Obesity status**						
no evidence of obesity	378,007 (78%)	379,686 (95%)	507,844 (80%)	95,815 (74%)	67,576 (93%)	100,597 (77%)
obese class I (30-34.9)	71,689 (15%)	12,352 (3.1%)	84,046 (13%)	21,422 (17%)	3,521 (4.8%)	19,933 (15%)
obese class II (35-39.9)	23,995 (4.9%)	3,889 (1.0%)	26,614 (4.2%)	7,960 (6.2%)	1,173 (1.6%)	6,806 (5.2%)
obese class III (40+)	11,466 (2.4%)	1,927 (0.5%)	12,563 (2.0%)	4,209 (3.3%)	599 (0.8%)	3,406 (2.6%)
**Body Mass Index (BMI)**	25.8 (22.9-29.4)	25.0 (22.2-28.4)	25.4 (22.7-28.9)	26.3 (23.3-30.1)	25.4 (22.5-29.2)	26.0 (23.0-29.6)
Unknown	0	295,893	0	0	48,039	0
**Harmful alcohol use**	21,816 (4.5%)	7,133 (1.8%)	18,470 (2.9%)	7,956 (6.1%)	2,332 (3.2%)	5,099 (3.9%)
**Smoking status (imputed)**						
Non-Smoker	247,979 (51%)	139,963 (57%)	344,413 (55%)	53,774 (42%)	25,393 (49%)	60,040 (46%)
Current Smoker	92,538 (19%)	57,998 (24%)	131,386 (21%)	32,237 (25%)	15,405 (30%)	35,017 (27%)
Ex-Smoker	128,631 (27%)	37,113 (15%)	140,366 (22%)	38,315 (30%)	9,332 (18%)	32,303 (25%)
Current Or Ex-Smoker	16,009 (3.3%)	9,255 (3.8%)	14,902 (2.4%)	5,080 (3.9%)	1,900 (3.7%)	3,382 (2.6%)
Unknown	0	153,525	0	0	20,839	0

[1] Median (IQR) or Frequency (%)

Table 9: Hazard ratios from Cox regression models for the association between inflammatory skin conditions and anxiety or depression. Full parameter estimates from all models are available in the supplementary materials (Table 10 - Table 13)

	Minimally adjusted model1				Confounder adjusted model2				Mediator adjusted model3			
**Exposure**	**N (total)**	**No. events/person-years (mil)**	**HR4**	**95% CI4**	**N**	**No. events/person-years (mil)**	**HR4**	**95% CI4**	**N**	**No. events/person-years (mil)**	**HR4**	**95% CI4**
**Atopic eczema**												
Anxiety												
Unexposed	3,720,478	263,430/22.3	-	-	3,605,107	248,322/21.5	-	-	3,182,332	223,563/19.9	-	-
Eczema	863,986	81,381/5.44	1.27	1.26-1.28	836,425	76,813/5.23	1.23	1.22-1.24	789,267	71,287/5.1	1.14	1.13-1.15
Depression												
Unexposed	3,228,884	323,857/19.1	-	-	3,122,038	307,557/18.3	-	-	2,722,547	273,009/16.8	-	-
Eczema	793,296	103,116/4.88	1.25	1.24-1.26	766,822	98,088/4.68	1.2	1.19-1.21	719,641	90,042/4.56	1.11	1.1-1.12
**Psoriasis**												
Anxiety												
Unexposed	1,365,258	103,326/9.69	-	-	1,307,447	95,615/9.2	-	-	1,175,868	86,597/8.64	-	-
Psoriasis	310,081	28,385/2.17	1.24	1.22-1.26	296,538	26,161/2.05	1.23	1.22-1.25	279,284	23,847/2	1.16	1.14-1.18
Depression												
Unexposed	1,177,299	126,748/8.3	-	-	1,125,173	118,217/7.86	-	-	1,001,225	106,058/7.34	-	-
Psoriasis	282,866	38,377/1.94	1.32	1.3-1.34	270,209	35,719/1.84	1.31	1.29-1.32	252,927	32,190/1.78	1.21	1.19-1.22

1 Adjusted for matched set (age, sex, GP)
2 Additionally adjusted for calendar period and comorbidities
3 Additionally adjusted for BMI, alcohol misuse, smoking status and (eczema only) sleep problems and steroid use
4 HR: Hazard ratio, CI: Confidence Interval

Table 10: All parameter estimates from Cox modelling of the association between eczema and anxiety. Three models were developed for each exposure-outcome association with varying levels of adjustment. The crude model adjusted for matched set only (age, sex, GP). The confounder adjusted model additionally adjusted for deprivation, calendar period, asthma (in eczema) or psoriatic arthropathy (in psoriasis), and Charlson comorbidity index. The mediator adjusted model additionally adjusted for BMI (as evidence of obesity), smoking status, harmful alcohol use. Mediator adjusted models in the eczema cohort additionally adjusted for sleep problems and immediate risk following a prescription of high dose oral glucocorticoids. Note: these estimates are presented for completeness and to aid reproducibility, The effect estimates for variables other than the primary exposure (eczema/psoriasis) carry no clinical relevance.

	Crude			Confounder			Mediator		
**Characteristic**	**HR[1,2]**	**95% CI[2]**	**p-value**	**HR[3,2]**	**95% CI[2]**	**p-value**	**HR[4,2]**	**95% CI[2]**	**p-value**
**Exposure**									
Unexposed	—	—		—	—		—	—	
Eczema	1.27	1.26, 1.28	<0.001	1.23	1.22, 1.24	<0.001	1.14	1.13, 1.15	<0.001
**Carstairs index of deprivation**									
1 (least deprived)				—	—		—	—	
2				1.08	1.06, 1.10	<0.001	1.05	1.03, 1.07	<0.001
3				1.16	1.14, 1.19	<0.001	1.11	1.09, 1.13	<0.001
4				1.25	1.23, 1.28	<0.001	1.15	1.13, 1.18	<0.001
5 (most deprived)				1.32	1.29, 1.35	<0.001	1.19	1.16, 1.22	<0.001
**Calendar Period**									
1997-2002				—	—		—	—	
2002-08				1.03	1.02, 1.05	<0.001	1.08	1.06, 1.09	<0.001
2008-14				1.01	0.99, 1.02	0.3	1.14	1.12, 1.16	<0.001
2014-19				1.18	1.16, 1.20	<0.001	1.35	1.32, 1.37	<0.001
**Asthma**									
No				—	—		—	—	
Yes				1.22	1.20, 1.23	<0.001	1.2	1.18, 1.22	<0.001
**Charlson's comorbidity index**									
Low				—	—		—	—	
Moderate				1.11	1.10, 1.13	<0.001	1.05	1.03, 1.06	<0.001
Severe				1.23	1.20, 1.27	<0.001	1.11	1.07, 1.15	<0.001
**Obesity**									
no evidence of obesity							—	—	
obese class I (30-34.9)							1	0.99, 1.02	0.7
obese class II (35-39.9)							1.05	1.03, 1.07	<0.001
obese class III (40+)							1.08	1.05, 1.11	<0.001
**Alcohol misuse**									
No							—	—	
Yes							1.59	1.55, 1.62	<0.001
**Smoking status**									
Non-Smoker							—	—	
Current Smoker							1.37	1.36, 1.39	<0.001

Ex-Smoker	1.19	1.18, 1.21	<0.001
Current Or Ex-Smoker	1.39	1.36, 1.43	<0.001
**Sleep problems**			
No	—	—	
Yes	2.41	2.39, 2.44	<0.001
**Recent high dose glucocorticoid steroid use (<30days)**			
No	—	—	
Yes	1.37	1.33, 1.41	<0.001

[1] Adjusted for matched set (age, sex, GP)
[2] HR = Hazard Ratio, CI = Confidence Interval
[3] Additionally adjusted for calendar period and comorbidities
[4] Additionally adjusted for BMI, alcohol misuse, smoking status, sleep problems, oral glucocorticoid use

*Table 11: All parameter estimates from Cox modelling of the association between **eczema and depression**. Three models were developed for each exposure-outcome association with varying levels of adjustment. The crude model adjusted for matched set only (age, sex, GP). The confounder adjusted model additionally adjusted for deprivation, calendar period, asthma (in eczema) or psoriatic arthropathy (in psoriasis), and Charlson comorbidity index. The mediator adjusted model additionally adjusted for BMI (as evidence of obesity), smoking status, harmful alcohol use. Mediator adjusted models in the eczema cohort additionally adjusted for sleep problems and immediate risk following a prescription of high dose oral glucocorticoids*
Note: these estimates are presented for completeness and to aid reproducibility, The effect estimates for variables other than the primary exposure (eczema/psoriasis) carry no clinical relevance.

**Eczema and depression**		**Crude**			**Confounder**			**Mediator**	
**Characteristic**	**HR[1,2]**	**95% CI[2]**	**p-value**	**HR[3,2]**	**95% CI[2]**	**p-value**	**HR[4,2]**	**95% CI[2]**	**p-value**
**Exposure**									
Unexposed	—	—		—	—		—	—	
Eczema	1.25	1.24, 1.26	<0.001	1.2	1.19, 1.21	<0.001	1.11	1.10, 1.12	<0.001
**Carstairs index of deprivation**									
1 (least deprived)				—	—		—	—	
2				1.14	1.12, 1.16	<0.001	1.11	1.10, 1.13	<0.001
3				1.29	1.26, 1.31	<0.001	1.22	1.20, 1.25	<0.001
4				1.46	1.44, 1.49	<0.001	1.33	1.31, 1.36	<0.001
5 (most deprived)				1.67	1.63, 1.70	<0.001	1.48	1.45, 1.52	<0.001
**Calendar Period**									
1997-2001				—	—		—	—	
2002-07				1.07	1.06, 1.08	<0.001	1.15	1.13, 1.16	<0.001
2008-13				1.1	1.09, 1.12	<0.001	1.31	1.29, 1.33	<0.001
2014-19				1.06	1.04, 1.07	<0.001	1.29	1.27, 1.31	<0.001
**Asthma**									

No	—	—		—	—	
Yes	1.07	1.05, 1.08	<0.001	1.05	1.04, 1.07	<0.001
**Charlson comorbidity index**						
Low (0)	—	—		—	—	
Moderate (1-2)	1.38	1.36, 1.40	<0.001	1.3	1.29, 1.32	<0.001
Severe (3+)	1.99	1.95, 2.04	<0.001	1.81	1.76, 1.85	<0.001
**Obesity**						
no evidence of obesity				—	—	
obese class I (30-34.9)				1.23	1.21, 1.24	<0.001
obese class II (35-39.9)				1.4	1.37, 1.43	<0.001
obese class III (40+)				1.58	1.53, 1.62	<0.001
**Alcohol misuse**						
No				—	—	
Yes				1.59	1.56, 1.63	<0.001
**Smoking status**						
Non-Smoker				—	—	
Current Smoker				1.64	1.62, 1.65	<0.001
Ex-Smoker				1.28	1.27, 1.30	<0.001
Current Or Ex-Smoker				1.66	1.62, 1.71	<0.001
**Sleep problems**						
No				—	—	
Yes				2.54	2.51, 2.57	<0.001
**Recent high dose glucocorticoid steroid use (<30days)**						
No				—	—	
Yes				1.39	1.35, 1.43	<0.001

[1] Adjusted for matched set (age, sex, GP)
[2] HR = Hazard Ratio, CI = Confidence Interval
[3] Additionally adjusted for calendar period and comorbidities
[4] Additionally adjusted for BMI, alcohol misuse, smoking status, sleep problems, oral glucocorticoid use

*Table 12: All parameter estimates from Cox modelling of the association between **psoriasis and anxiety**. Three models were developed for each exposure-outcome association with varying levels of adjustment. The crude model adjusted for matched set only (age, sex, GP). The confounder adjusted model additionally adjusted for deprivation, calendar period, asthma (in eczema) or psoriatic arthropathy (in psoriasis), and Charlson comorbidity index. The mediator adjusted model additionally adjusted for BMI (as evidence of obesity), smoking status, harmful alcohol use.*

Note: these estimates are presented for completeness and to aid reproducibility, The effect estimates for variables other than the primary exposure (eczema/psoriasis) carry no clinical relevance.

**Psoriasis and anxiety**		**Crude**			**Confounder**			**Mediator**	
**Characteristic**	**HR**[1,2]	**95% CI**[2]	**p-value**	**HR**[3,2]	**95% CI**[2]	**p-value**	**HR**[4,2]	**95% CI**[2]	**p-value**
**Exposure**									
Unexposed	—	—		—	—		—	—	
Psoriasis	1.24	1.22, 1.26	<0.001	1.23	1.22, 1.25	<0.001	1.16	1.14, 1.18	<0.001
**Carstairs index of deprivation**									
1 (least deprived)				—	—		—	—	
2				1.07	1.03, 1.10	<0.001	1.05	1.02, 1.08	0.003
3				1.13	1.10, 1.17	<0.001	1.09	1.06, 1.13	<0.001
4				1.23	1.19, 1.27	<0.001	1.16	1.12, 1.21	<0.001
5 (most deprived)				1.33	1.28, 1.39	<0.001	1.25	1.20, 1.30	<0.001
**Calendar Period**									
1997-2001				—	—		—	—	
2002-07				1.05	1.03, 1.08	<0.001	1.17	1.14, 1.20	<0.001
2008-13				1.04	1.01, 1.06	0.005	1.3	1.26, 1.33	<0.001
2014-19				1.15	1.12, 1.18	<0.001	1.48	1.43, 1.52	<0.001
**Psoriatic arthritis**									
No				—	—		—	—	
Yes				0.84	0.79, 0.90	<0.001	0.85	0.79, 0.92	<0.001
**Charlson comorbidity index**									
Low (0)				—	—		—	—	
Moderate (1-2)				1.28	1.26, 1.30	<0.001	1.25	1.23, 1.27	<0.001
Severe (3+)				1.47	1.40, 1.54	<0.001	1.41	1.35, 1.48	<0.001
**Obesity**									
no evidence of obesity							—	—	
obese class I (30-34.9)							1	0.98, 1.02	0.8
obese class II (35-39.9)							1.02	0.98, 1.05	0.3
obese class III (40+)							1.08	1.03, 1.14	<0.001
**Alcohol misuse**									

No							—	—	
Yes							1.74	1.69, 1.80	<0.001
**Smoking status**									
Non-Smoker							—	—	
Current Smoker							1.36	1.34, 1.39	<0.001
Ex-Smoker							1.19	1.16, 1.21	<0.001
Current Or Ex-Smoker							1.42	1.35, 1.48	<0.001

[1] Adjusted for matched set (age, sex, GP)
[2] HR = Hazard Ratio, CI = Confidence Interval
[3] Additionally adjusted for calendar period and comorbidities
[4] Additionally adjusted for BMI, alcohol misuse, smoking status

Table 13: All parameter estimates from Cox modelling of the association between ***psoriasis and depression****. Three models were developed for each exposure-outcome association with varying levels of adjustment. The crude model adjusted for matched set only (age, sex, GP). The confounder adjusted model additionally adjusted for deprivation, calendar period, asthma (in eczema) or psoriatic arthropathy (in psoriasis), and Charlson comorbidity index. The mediator adjusted model additionally adjusted for BMI (as evidence of obesity), smoking status, harmful alcohol use.*
Note: these estimates are presented for completeness and to aid reproducibility, The effect estimates for variables other than the primary exposure (eczema/psoriasis) carry no clinical relevance.

**Psoriasis and depression**		**Crude**			**Confounder**			**Mediator**	
**Characteristic**	**HR**[1,2]	**95% CI**[2]	**p-value**	**HR**[3,2]	**95% CI**[2]	**p-value**	**HR**[4,2]	**95% CI**[2]	**p-value**
**Exposure**									
Unexposed	—	—		—	—		—	—	
Psoriasis	1.32	1.30, 1.34	<0.001	1.31	1.29, 1.32	<0.001	1.21	1.19, 1.22	<0.001
**Carstairs index of deprivation**									
1 (least deprived)				—	—		—	—	
2				1.16	1.13, 1.20	<0.001	1.15	1.12, 1.18	<0.001
3				1.32	1.28, 1.35	<0.001	1.26	1.23, 1.30	<0.001
4				1.53	1.49, 1.58	<0.001	1.42	1.37, 1.47	<0.001
5 (most deprived)				1.76	1.70, 1.82	<0.001	1.57	1.51, 1.64	<0.001
**Calendar Period**									
1997-2001				—	—		—	—	
2002-07				1.06	1.04, 1.08	<0.001	1.19	1.17, 1.22	<0.001

2008-13	1.12	1.09, 1.14	<0.001	1.44	1.40, 1.47	<0.001
2014-19	1.01	0.98, 1.03	0.5	1.35	1.31, 1.38	<0.001
**Psoriatic arthritis**						
No	—	—		—	—	
Yes	1.08	1.02, 1.15	0.008	1.11	1.04, 1.18	0.001
**Charlson comorbidity index**						
Low (0)	—	—		—	—	
Moderate (1-2)	1.47	1.45, 1.49	<0.001	1.42	1.40, 1.44	<0.001
Severe (3+)	2.05	1.98, 2.13	<0.001	1.97	1.89, 2.05	<0.001
**Obesity**						
no evidence of obesity				—	—	
obese class I (30-34.9)				1.21	1.19, 1.23	<0.001
obese class II (35-39.9)				1.34	1.30, 1.38	<0.001
obese class III (40+)				1.56	1.50, 1.63	<0.001
**Alcohol misuse**						
No				—	—	
Yes				1.65	1.60, 1.70	<0.001
**Smoking status**						
Non-Smoker				—	—	
Current Smoker				1.59	1.56, 1.61	<0.001
Ex-Smoker				1.25	1.23, 1.27	<0.001
Current Or Ex-Smoker				1.63	1.56, 1.70	<0.001

[1] Adjusted for matched set (age, sex, GP)

[2] HR = Hazard Ratio, CI = Confidence Interval

[3] Additionally adjusted for calendar period and comorbidities

[4] Additionally adjusted for BMI, alcohol misuse, smoking status

Table 14: Analysis of individual with/without a consultation with their GP either 1-year or 3-years before study entry, comparing those with and without the inflammatory skin condition of interest

Exposure status	*n* without a recent consultation	%	*n* with a recent consultation	%
People with/without a consultation < 1 year before study entry				
**Psoriasis cohort**				
Unexposed	435,654	23.8	1,398,676	76.2
Psoriasis	34,212	9.3	332,672	90.7
**Eczema cohort**				
Unexposed	1,129,891	22.6	3,860,234	77.4
Eczema	87,968	8.5	944,814	91.5
People with/without a consultation < 3 years before study entry				
**Psoriasis cohort**				
Unexposed	206,798	11.3	1,627,532	88.7
Psoriasis	18,788	5.1	348,096	94.9
**Eczema cohort**				
Unexposed	511,951	10.3	4,478,174	89.7
Eczema	36,427	3.5	996,355	96.5

Table 15: Estimates of the time-varying hazard ratios for the association between eczema/psoriasis and anxiety/depression at multiple time-points following study entry. All models adjusted for age, sex, GP, deprivation, calendar period, asthma (in eczema) or psoriatic arthropathy (in psoriasis), and Charlson comorbidity index, BMI (as evidence of obesity), smoking status, harmful alcohol use (plus sleep problems and high dose oral glucocorticoid use in the eczema models). Confidence intervals for the spline models show the 2.5 and 97.5 percentiles from 200 bootstrap iterations of the model.

	**At study entry**	**+ 1 year**	**+ 3 years**	**+ 5 years**
Eczema				
Anxiety	1.13 (1.10-1.16)	1.17 (1.16-1.19)	1.12 (1.09-1.14)	1.10 (1.05-1.15)
Depression	1.09 (1.06-1.11)	1.14 (1.12-1.16)	1.09 (1.07-1.12)	1.07 (1.02-1.12)
Psoriasis				
Anxiety	1.22 (1.17-1.26)	1.19 (1.15-1.22)	1.13 (1.08-1.17)	1.07 (0.99-1.14)
Depression	1.25 (1.20-1.30)	1.24 (1.21-1.27)	1.16 (1.12-1.20)	1.18 (1.12-1.25)

Table 16: Number of people meeting our eczema algorithm, psoriasis definition or both in the overall exposed cohorts

	Skin disease diagnoses						
Skin disease cohort	One only (%)		Eczema -> psoriasis (%)		Psoriasis -> eczema (%)		Total
Eczema	952,702	92.25	53,895	5.22	26,185	2.54	1,032,782
Psoriasis	286,804	78.17	53,895	14.69	26,185	7.14	366,884

Supplementary figures

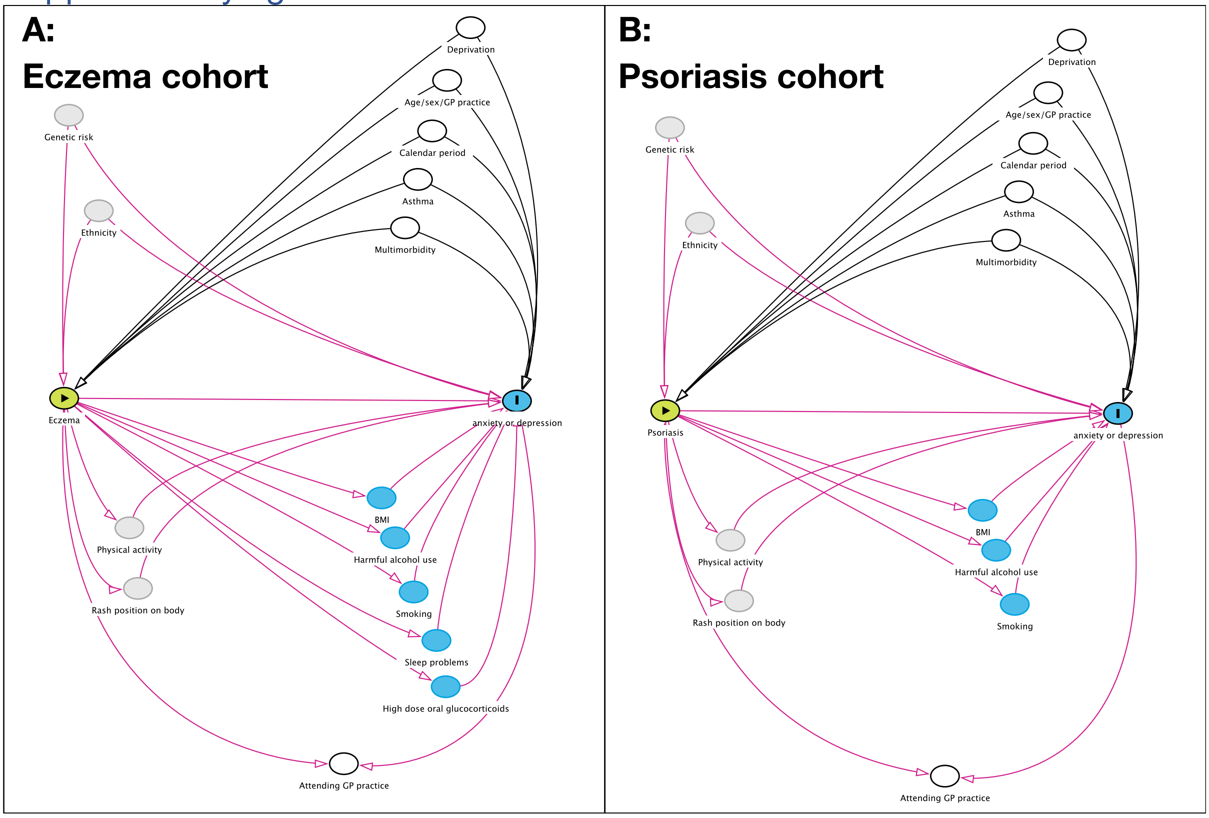

Figure 1: Directed acyclic graph for the association between eczema or psoriasis with anxiety or depression. White and blue circles, variables adjusted for in this study. Grey circles, unobserved variables not included in models (except ethnicity which is included in a sensitivity analysis). For simplicity we have not displayed possible links between covariates (e.g., BMI and harmful alcohol use)

Supplementary Figure 1 shows directed acyclic graphs (DAGs) to illustrate our presumed causal relationships (based on existing literature and clinical experience) between the primary exposures in this study (atopic eczema or psoriasis) and the outcome (new onset anxiety or depression). We used these DAGs to identify potential variables for use in the study (and classify them as either: 1) confounders – a common cause of both exposure and outcome; or 2) mediators – caused by the exposure and a possible cause of the outcome) and consequently inform our modelling strategy. Our DAGs also demonstrate that there are some factors that are unmeasured in our main analyses (genetic risk, ethnicity, physical activity, rash position on body) as we could not capture them robustly in primary care data, as well as potential collider bias because both exposure and outcome are causes of selection in the data (attending GP practice). These DAGs were drawn using https://http://www.dagitty.net [20].

http://www.dagitty.net/

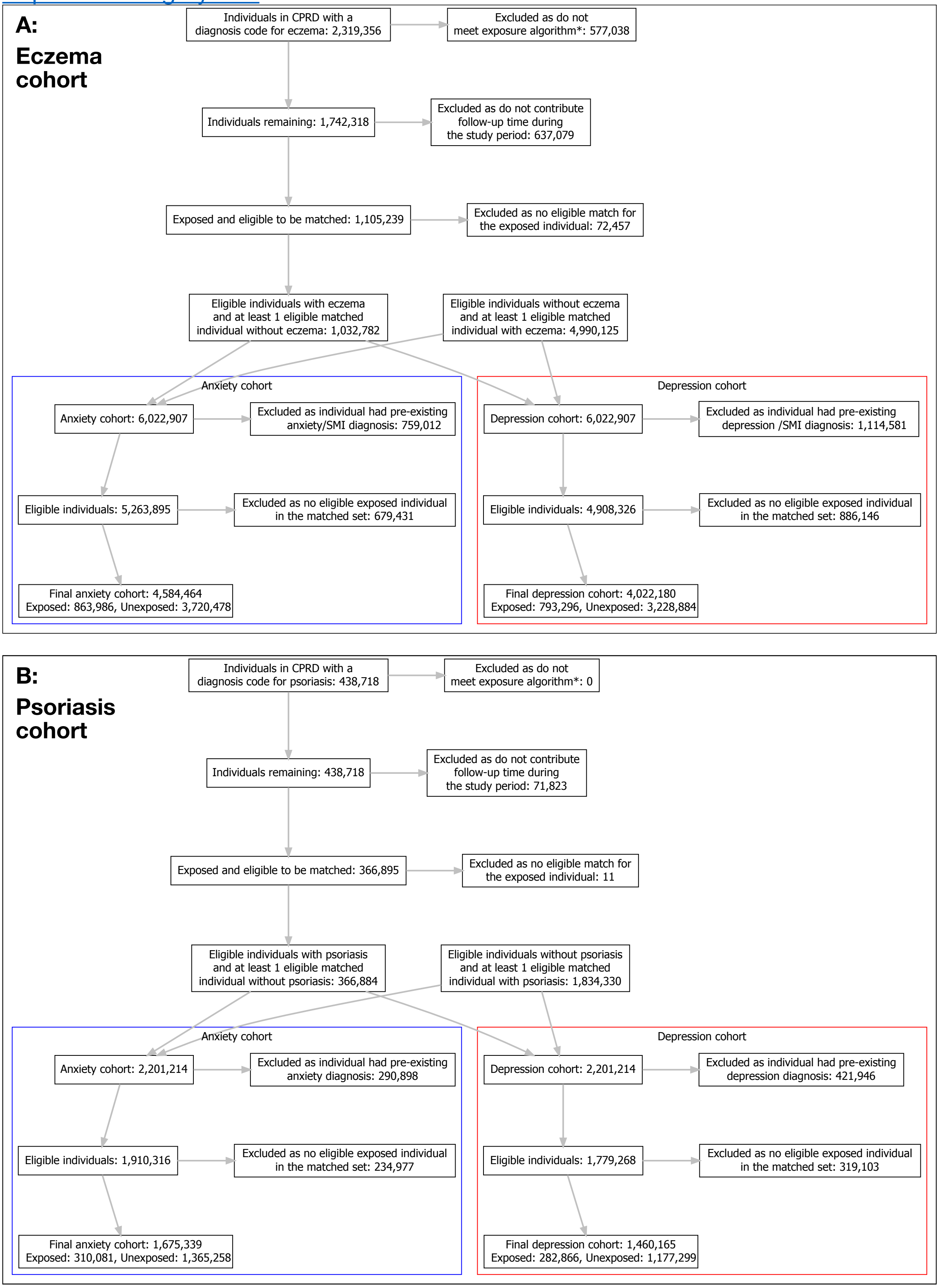

*Figure 2: Participant flowcharts. * exposure algorithms: eczema (based on 1x diagnostic code and 2x therapy records on separate days); psoriasis (based on 1x diagnostic code)*

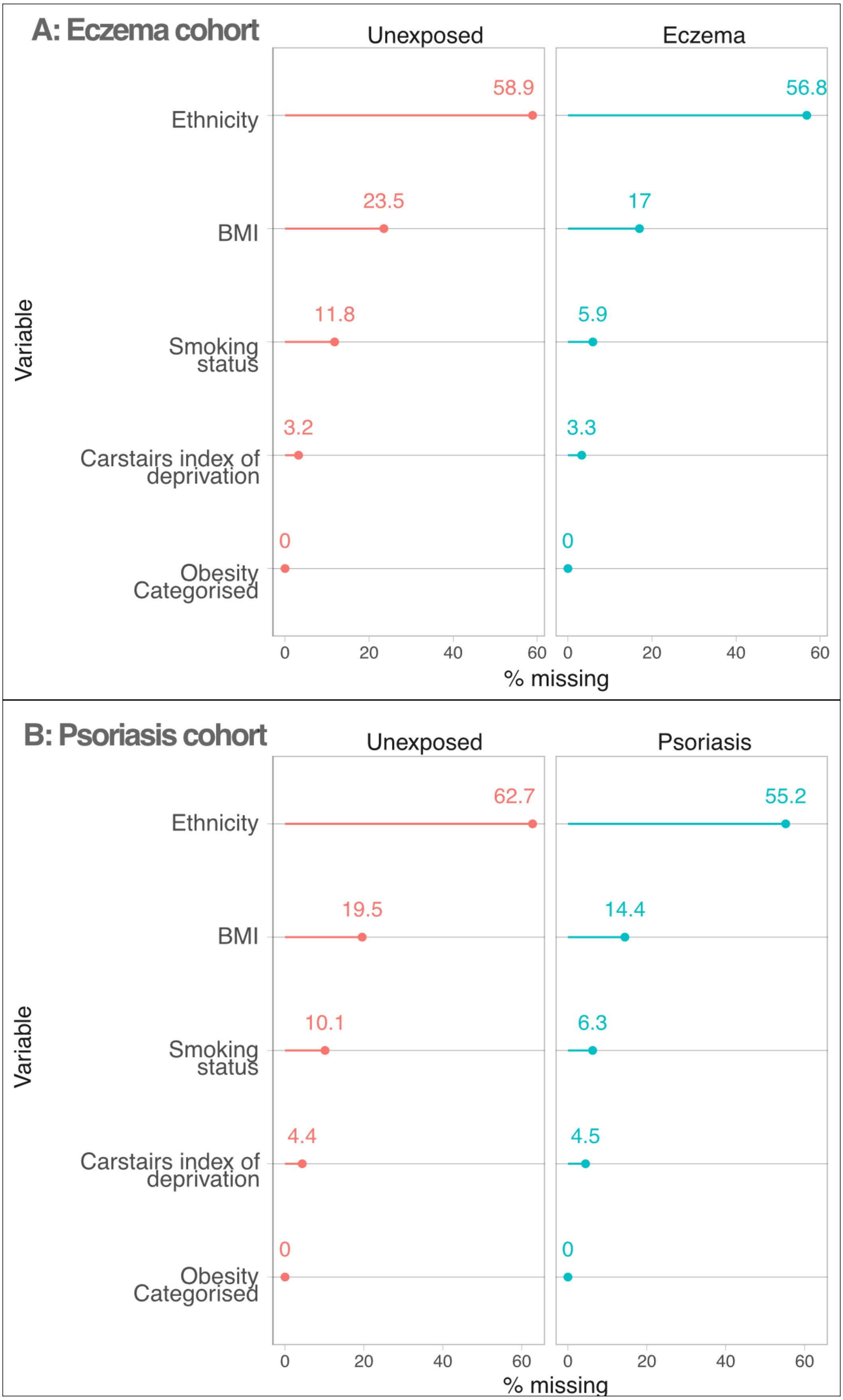

Figure 3: Percentage of missing data in the both the eczema (A) and psoriasis (B) cohorts, comparing those with and without eczema/psoriasis

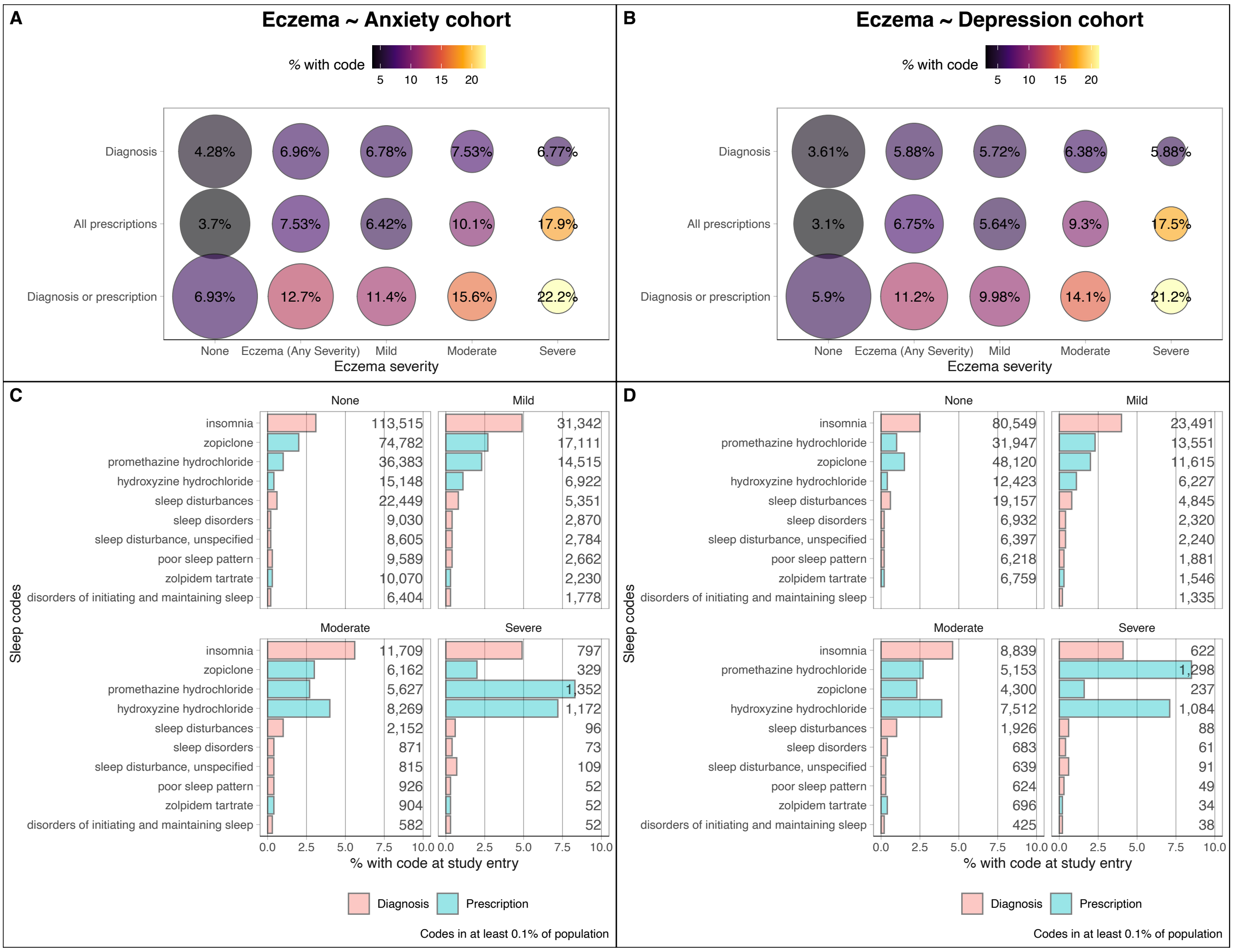

Figure 4: Percentage of individuals with eczema at study entry with a record for a diagnosis or prescription for sleeping problems by varying levels of eczema severity. (A and B) percentage of the cohort with a record of a sleep problems diagnosis or sleep-specific prescriptions. Size of bubble is scaled by number of codes in each group. Colour and label show the percentage with a code at baseline. (B) Percentage of cohort with specific treatments/diagnoses by eczema severity. All codes with at least 0.1% prevalence over the study period are displayed

Supplementary Figure 4 shows that medication for sleeping problems and diagnoses for insomnia are more common in those with eczema compared to matched comparators. Panels A and B also demonstrate that as eczema severity increases, the proportion of people with at least one diagnosis or prescription for sleeping problems increases, from 11.4% in those with mild eczema to 22.2% in those with severe eczema (in the anxiety cohort). Panels C and D highlight the common prescriptions and diagnoses for sleeping problems in our cohorts, zopiclone was the most common medication prescribed overall, however in the severe eczema group promethazine and hydroxyzine are the most common prescriptions.

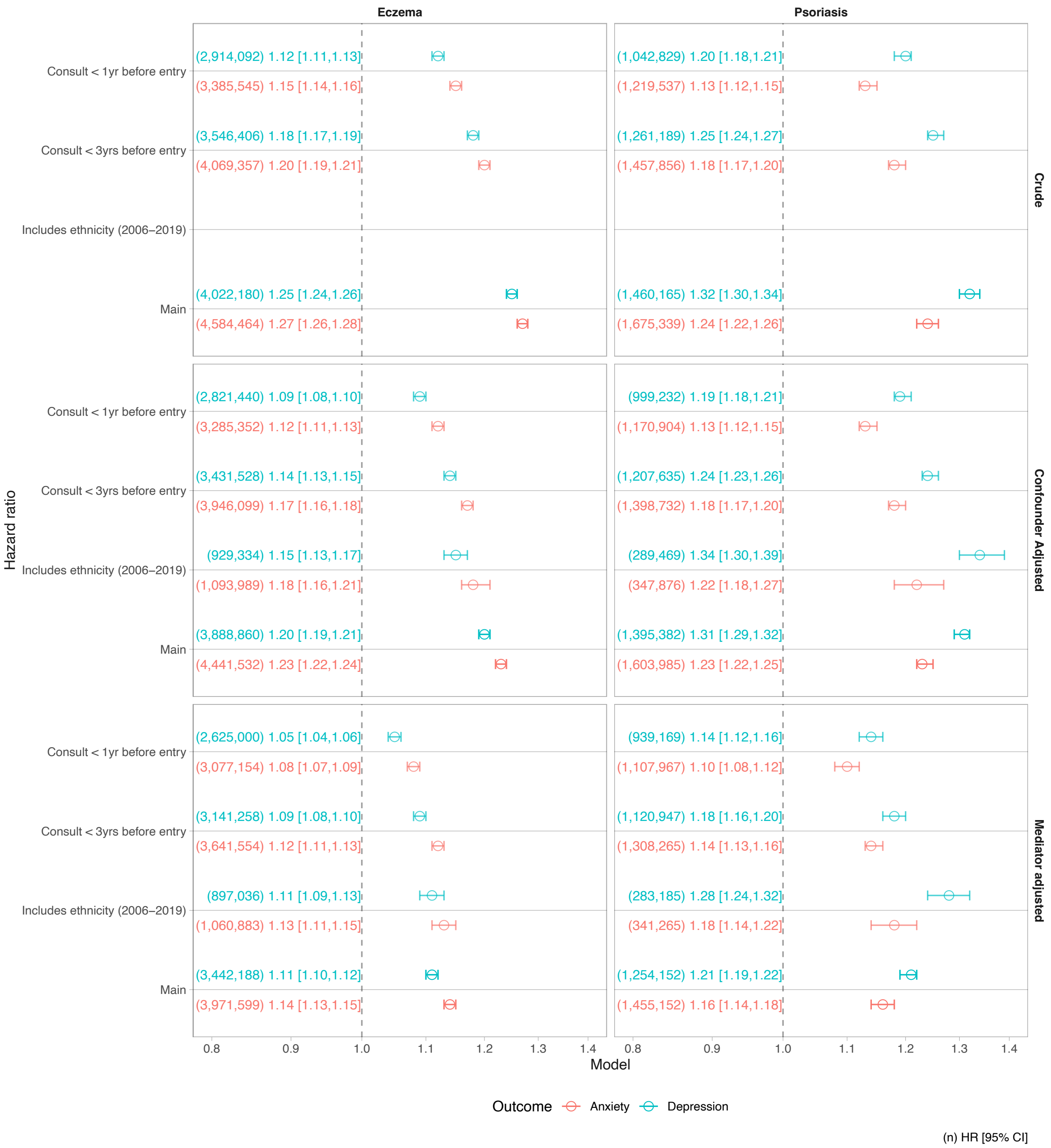

Figure 5: Sensitivity analyses: Hazard ratios (HRs) for the association between inflammatory skin conditions. and anxiety or depression from stratified Cox models. Three models were developed for each exposure-outcome association with varying levels of adjustment. The crude model adjusted for matched set only (age, sex, GP). The confounder adjusted model additionally adjusted for deprivation, calendar period, asthma (in eczema) or psoriatic arthropathy (in psoriasis), and Charlson comorbidity index. The mediator adjusted model additionally adjusted for BMI (as evidence of obesity), smoking status, harmful alcohol use. Mediator adjusted models in the eczema cohort additionally adjusted for sleep problems and immediate risk following a prescription of high dose oral glucocorticoids. Dots; estimated HR. Lines; 95% CI. Text shows (the number of individuals in the model) HR, [95% CI].

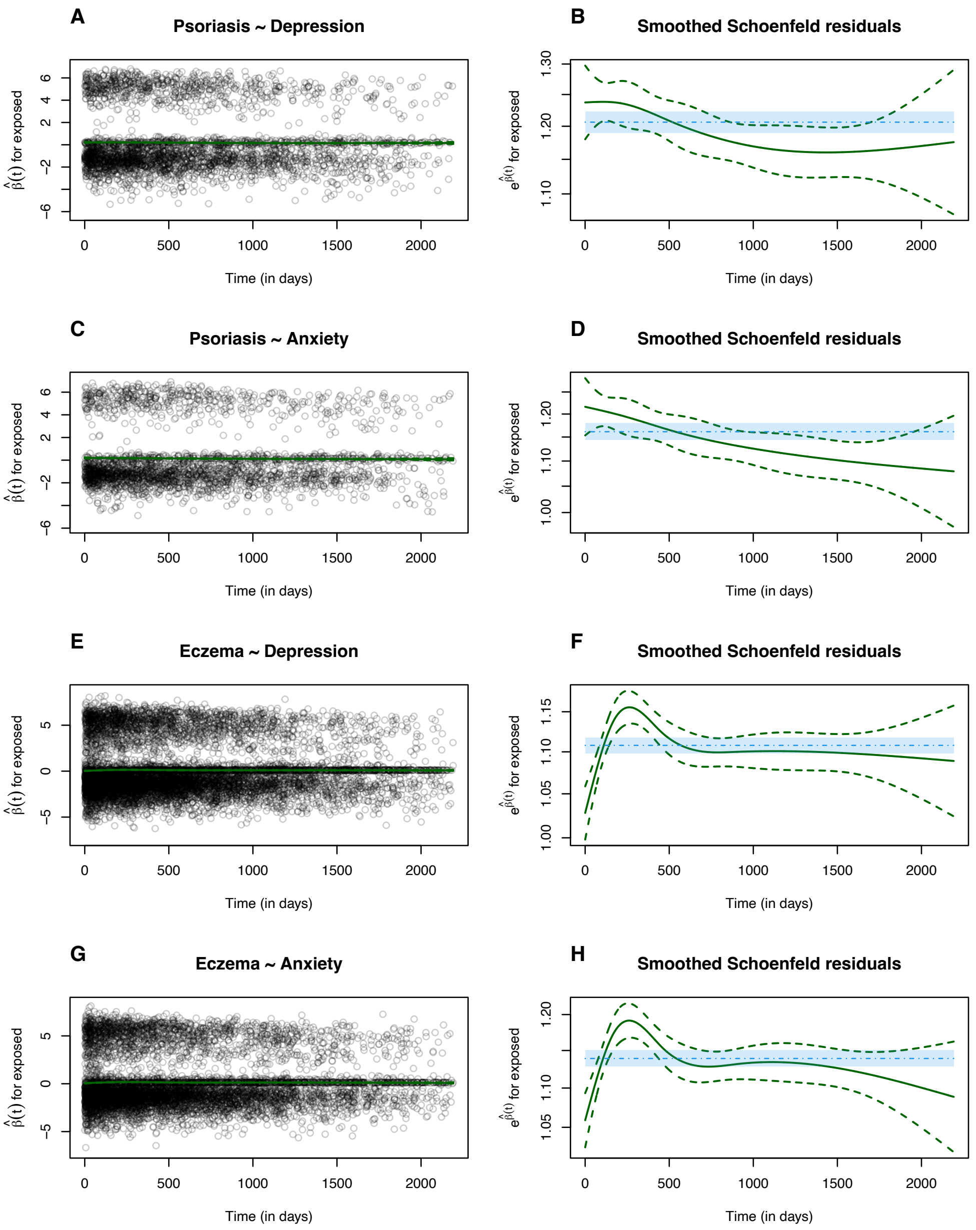

Figure 6: Checking the proportional hazards assumption for the association between eczema /psoriasis and anxiety/depression. Left column, scaled Schoenfeld residuals from the mediator-adjusted Cox model. Right column, zoomed in on the smoothed spline through the scaled Schoenfeld residuals in left-hand panel, which is equivalent to a time varying HR from the model. Green solid and dashed lines, median estimate and 95% CI; Blue dotted line and region, the corresponding proportional HR from the Cox model and 95% CI.

Supplementary Figure 6 shows the plots of scaled Schoenfeld residuals from the main analysis to assess the proportional hazards assumption. All models adjusted for age, sex, GP, deprivation, calendar period, asthma (in eczema) or psoriatic arthropathy (in psoriasis), and Charlson comorbidity index, BMI (as evidence of obesity), smoking status, harmful alcohol use (plus sleep problems and high dose oral glucocorticoid use in the eczema models). If the proportional hazards assumption is valid, the smoothed estimate through the Schoenfeld residuals should not vary over time. Residuals were calculated using the `*cox.zph*` function from the Survival package in R [54].

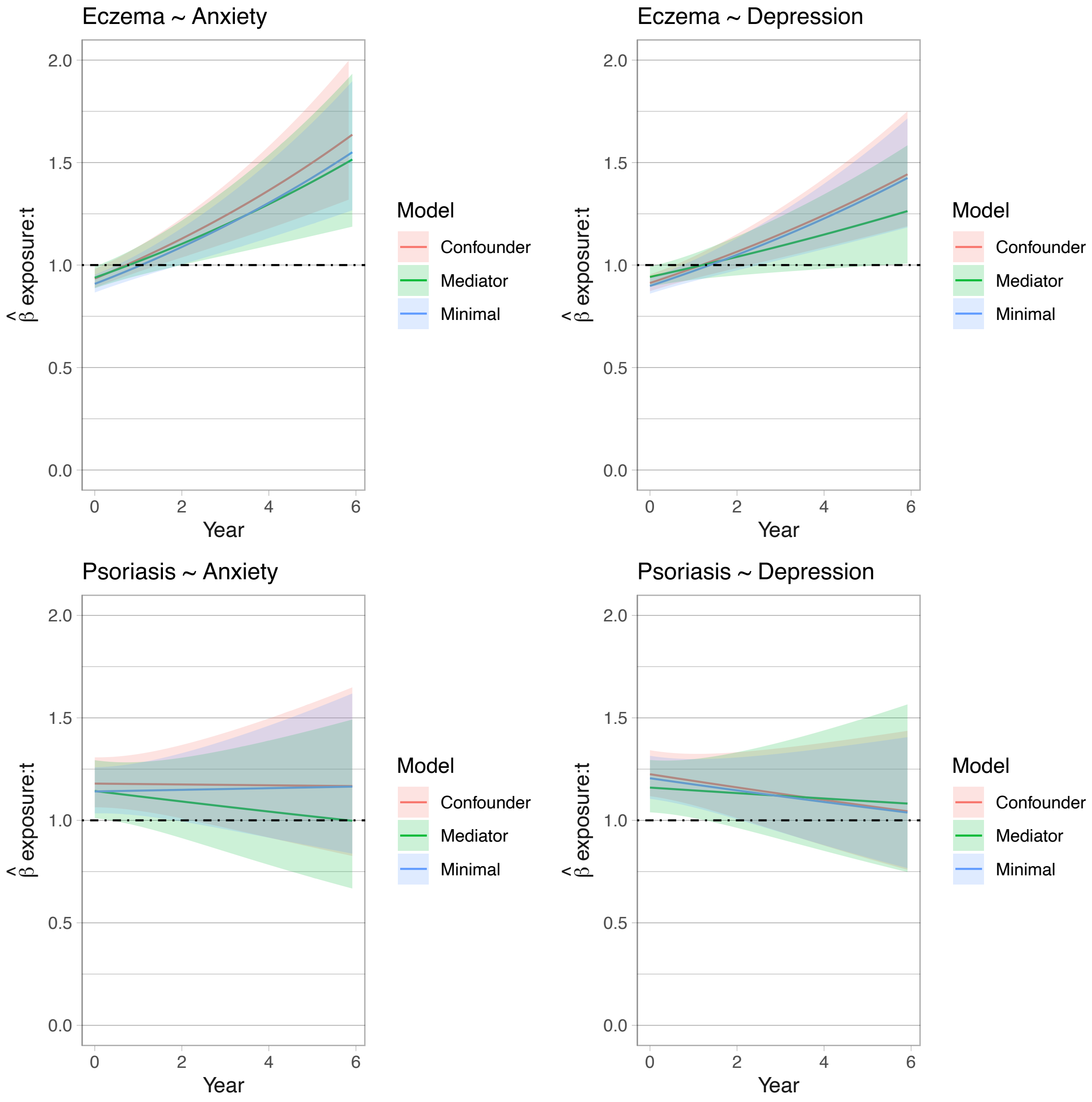

Figure 7: Exploring non-constant hazard ratios (HRs) for the association between inflammatory skin conditions and anxiety or depression by including a linear interaction with time. The assumption that time acts in a linear fashion is strong and lead to extreme results in later periods of follow up. These results were improved on by including a non-linear interaction with time in the main paper

Supplementary Figure 7 shows the estimated hazard ratio from a Cox model including an interaction between the primary exposure (eczema/psoriasis) and time since study entry. These models allow for time-varying hazard ratios but are restricted to linear functions of time and therefore the penalised spline method was preferred and presented in the main manuscript (Figure 3).

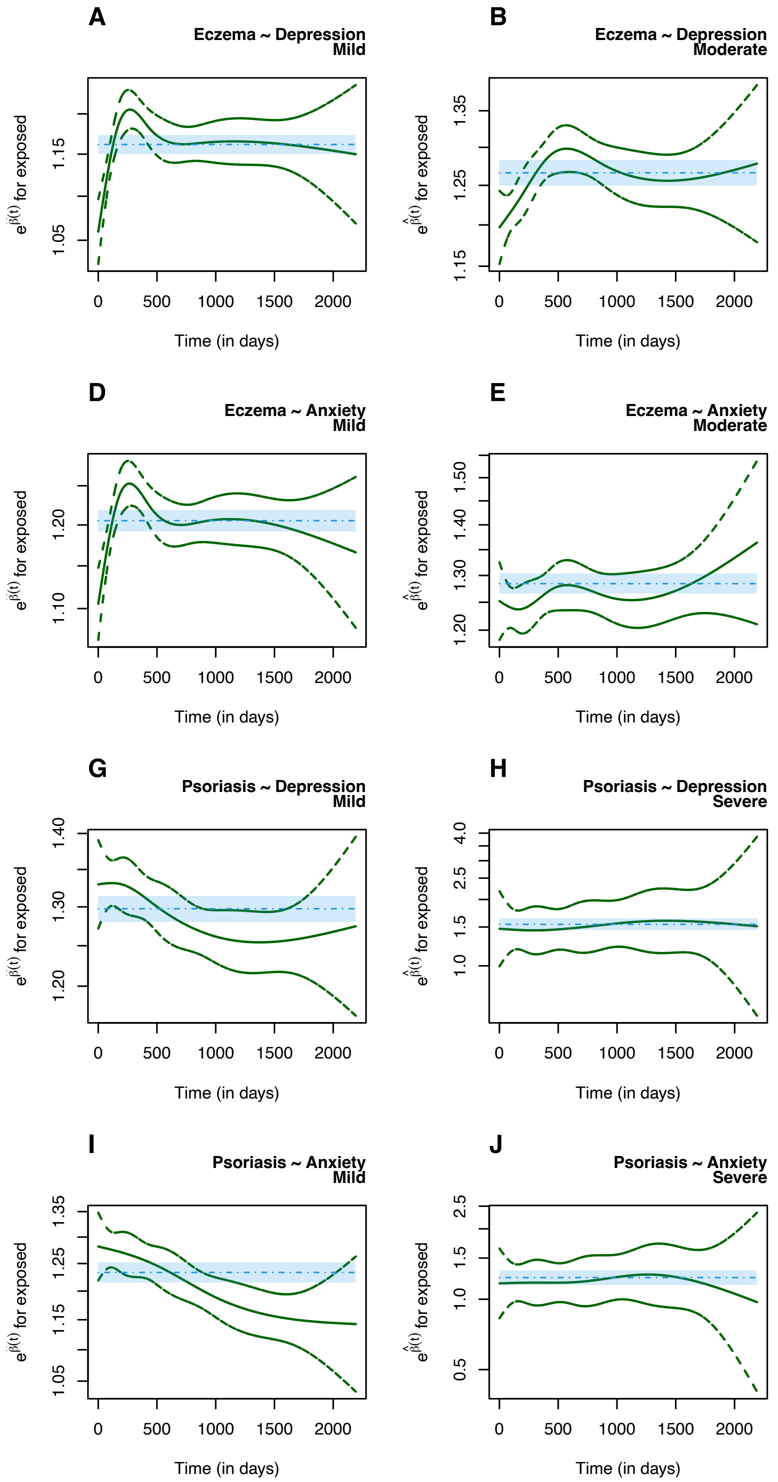

*Figure 8: Checking the proportional hazards assumption for the association between **eczema/psoriasis severity and anxiety/depression**. Each row shows estimates from a single Cox model. Plots show a smoothed spline through the exponential of the scaled Schoenfeld residuals which is equivalent to a time varying HR from the model. Green solid and dashed lines, median estimate and 95% CI; Blue dotted line and region, the corresponding proportional HR from the Cox model and 95% CI. All models adjusted for age, sex, GP, deprivation, calendar period, asthma (in eczema) or psoriatic arthropathy (in psoriasis), and Charlson comorbidity index, BMI (as evidence of obesity), smoking status, harmful alcohol use (plus sleep problems and high dose oral glucocorticoid use in the eczema models).*

Supplementary Figure 8 shows the smoothed average scaled Schoenfeld residuals from four Cox models estimating the effect of eczema or psoriasis severity on the

hazard of anxiety or depression to assess the proportional hazards assumption. For models of eczema severity there are 3 levels of severity (mild, moderate, severe) and for models of psoriasis severity there are two levels (mild, severe). If the proportional hazards assumption is valid, the smoothed estimate through the Schoenfeld residuals should not vary over time. These plots suggest that as skin condition severity increases, the proportional hazards assumption is more likely to be valid (horizontal average Schoenfeld residuals) but in groups with the mildest form of skin conditions there is a period of elevated hazard in the first 1-2 years after study entry.

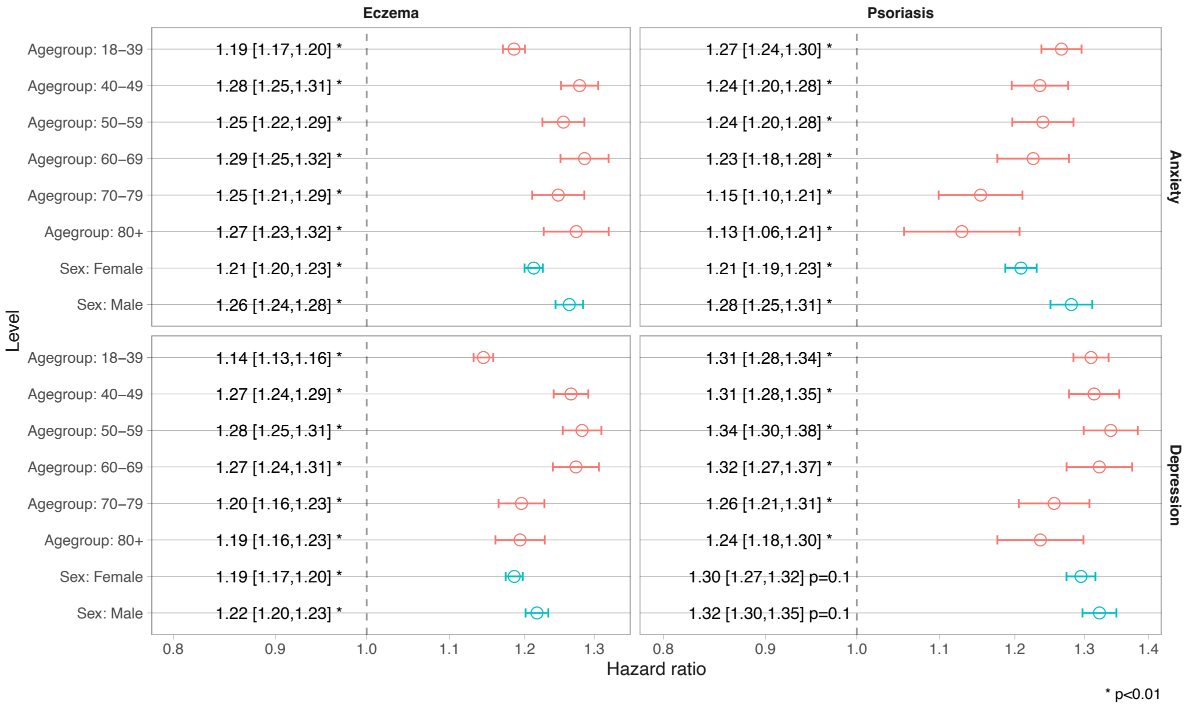

Figure 9: Estimated hazard ratios for the total effect of inflammatory skin conditions on anxiety or depression, modified by several pre-specified effect modifiers. HRs and 95% confidence intervals are derived from linear combinations of model parameters (confounder) adjusted for age, sex, GP, deprivation, calendar period, asthma (in eczema) or psoriatic arthropathy (in psoriasis), and Charlson comorbidity index. Dots; estimated HR. Lines; 95% CI
